# Apigenin and its combination with Vorinostat induces apoptotic-mediated cell death in TNBC by modulating the epigenetic and apoptotic regulators and related miRNAs

**DOI:** 10.1038/s41598-024-60395-x

**Published:** 2024-04-25

**Authors:** Snehal Nimal, Navanath Kumbhar, Saruchi, Shriya Rathore, Nitin Naik, Sneha Paymal, Rajesh N. Gacche

**Affiliations:** 1 Department of Biotechnology, Savitribai Phule Pune University (SPPU), Pune, 411007 Maharashtra (MS) India; 2 Medical Information Management, Department of Biochemistry, Shivaji University, Kolhapur, 416004 Maharashtra (MS) India; 3 Department of Microbiology, Shivaji University, Kolhapur, 416004 Maharashtra (MS) India

**Keywords:** TNBC, HDACs, miRNAs, Apoptosis, Flavonoids, Apigenin and MD simulations, Cancer, Computational biology and bioinformatics, Drug discovery

## Abstract

Triple-negative breast cancer (TNBC) is a metastatic disease and a formidable treatment challenge as it does not respond to existing therapies. Epigenetic regulators play a crucial role in the progression and metastasis by modulating the expression of anti-apoptotic, pro-apoptotic markers and related miRNAs in TNBC cells. We have investigated the anti-TNBC potential of dietary flavonoid ‘Apigenin’ and its combination with Vorinostat on MDA-MB-231 cells. At Apigenin generated ROS, inhibited cell migration, arrested the cell cycle at subG0/G1 phases, and induced apoptotic-mediated cell death. Apigenin reduced the expression of the class-I HDACs at the transcriptomic and proteomic levels. In the immunoblotting study, Apigenin has upregulated pro-apoptotic markers and downregulated anti-apoptotic proteins. Apigenin inhibited the enzymatic activity of HDAC/DNMT and increased HAT activity. Apigenin has manifested its effect on miRNA expression by upregulating the tumor-suppressor miR-200b and downregulation oncomiR-21. Combination study reduced the growth of TNBC cells synergistically by modulating the expression of epigenetic and apoptotic regulators. Molecular docking and MD simulations explored the mechanism of catalytic inhibition of HDAC1 and HDAC3 and supported the in-vitro studies. The overall studies demonstrated an anti-TNBC potential of Apigenin and may help to design an effective strategy to treat metastatic phenotype of TNBC.

## Introduction

The triple-negative breast cancer (TNBC) is a subgroup of breast cancer characterized by high metastatic growth and aggressive invasion ability with extensive inter- and intra-tumour heterogeneity^1,2^. Over 2.3 million cases of breast cancer were diagnosed and 685,000 cancer deaths were reported all over the world. TNBC accounts for 10–20% of all breast cancers and become a major cause of high female mortality across the globe^1–3^. TNBC does not express the progesterone receptor (PR), estrogen receptor (ER), and human epidermal growth factor receptor 2 (HER2)^4^. Due to this, TNBCs displayed a more aggressive phenotype that is associated with poor prognosis and exhibits acquired drug resistance towards the currently available targeted and hormonal therapies^5^. In the mainstream of carcinogenesis, epigenetic modifications including histone acetylation and histone deacetylation play a critical role in the progression and metastasis of TNBCs^6–8^. The acetylation of lysine residues on histone and non-histone proteins by histone acetyltransferase (HAT) resulted in the remodelling of chromatin which activates the transcription of genes involved in the cell cycle regulation in normal cells^8^. In contrast, the deacetylation of histone (H3/H4) and non-histone (p53, PTEN, Hsp90, Tubulin, and NH-α) proteins by histone deacetylases (HDACs) produces a more condensed chromatin state that alters the transcription of oncogenes and tumour suppressor genes^9–13^. This initiates abnormal cell proliferation, invasion, and metastasis in TNBC and other cancer subtypes^8,9,14^. HDACs are the class of Zn^2+^ (Class I, II, and IV) and NAD^+^-dependent (Class III) enzymes that participate in cancer progression and are attributed to aggressive tumour formation by modulating the regulation of microRNAs (miRNAs) in various human cancers^15–19^.

The role of HDACs in the modulation of cell cycle, cell proliferation, invasion, differentiation, angiogenesis, and metastasis has been extensively investigated in TNBCs using in-vitro and in-vivo studies^20–22^. The elevated HDAC level suppressed the expression of tumour suppressor genes and regulated various oncogenic pathways in TNBCs^22^. The high expression of HDAC1, HDAC2, and HDAC3 is involved in the initiation, progression, and more aggressive phenotype of breast and other cancer subtypes^23,24^. The increased HDAC3 phosphorylation reduced the binding ability and selectivity of HDAC inhibitors towards HDAC3 in MDA-MB-231 cells^25^. The HDAC4 deacetylate histone-3 in the promoter region of SMAD4 produces drug resistance in breast cancer cells against 5-fluorouracil^26^. HDAC5 has increased cell invasion, metastasis, stemness, and chemoresistance in MDA-MB-231 cells^22,27^. Similarly, the HDAC6 increased the expression of estrogen-related genes and enhanced the aggressiveness in breast cancer^28–30^. The high expressions of HDAC1 and HDAC7 were associated with the metastatic stage of TNBC cells^31^. The elevated expression of HDAC8 regulated the Hippo-YAP signals and increased the migration ability of TNBC cells^32^. Further, HDAC9 activated the VEGF and MAPK3 kinase pathways by modulating the miR-206 expression and enhanced the cell invasion and angiogenesis in TNBCs^33^. The HDAC10 reduced the overall survival rate in TNBC patients by decreasing the expression of ESR1^34^. On the same line, the high HDAC11 expression was correlated to the progressive metastatic condition of breast cancer^35^. Overall, the preceding literature reports explored the pathophysiological role of HDACs in the initiation, progression, and metastasis of TNBCs. Therefore, HDACs were considered promising therapeutic targets for designing potent anticancer agents^14,36^.

Many experimental and computational studies have been performed to control the progression of TNBC by targeting HDAC isoforms^20,24,36–42^. The HDAC inhibitors alone and in combination with other anticancer agents inhibited cell proliferation, invasion, and metastasis in TNBC and other cancer subtypes^36–42^. The HDACi (vorinostat, panobinostat, entinostat, and valproic acid) in combination with an immune checkpoint inhibitor (anti-PD-1 and anti-CTLA-4 blockades) induces apoptosis by activating the PD-1/PD-L1 pathways in TNBC cells and mice model^43^. Similarly, HDACi (vorinostat, panobinostat, romidepsin, and givinostat) in combination with EC359 (leukaemia inhibitory factor receptor inhibitor) and isaindigotone scaffold suppressed cell viability, colony formation, and invasion by inducing apoptosis in TNBC cells and xenograft tumour model^44,45^. The combinations of vorinostat, recolinostat, and OBP-801 with eribulin increased acetylation of α-tubulin by downregulating the anti-apoptotic Bcl2 and survivin proteins which subsequently suppress the MAPK pathway^46,47^. In addition, SAHA improved the anti-tumour potential of olaparib (PARP inhibitor) by increasing the expression of PTEN and downregulating the AKT, p-AKT, ERK, p-ERK, and p-STAT3 pathways^48^. A combination of SAHA with Pargyline (lysine-specific demethylase-1) induced apoptotic-mediated cell death in TNBC cells^49^. Further, SAHA reduced lung metastasis by enhancing the radio sensitivity of human and mouse TNBC cells^50^. Panobinostat suppressed the expression of ZEB family proteins involved in the EMT process and reduced mesenchymal phenotype and metastasis in TNBC cells along with claudin-low-TNBC patients^51,52^.

The series of above described in-vitro and in-vivo studies displayed the significant contribution of HDACi in inhibiting cell proliferation, invasion, and metastasis by combating drug resistance in TNBC. However, these drugs produced many severe side effects such as the reduction of white/red blood cells, hair loss, ulcers, changes in skin colour, and various hormonal changes^53–56^. Hence, there is an urgent need to develop new therapeutic strategies that are safer and more effective against TNBC^57,58^. In this direction, researchers have tested various natural flavonoids alone and in combination with standard HDACi as anti-cancer drugs that modulate the epigenetic changes as well as miRNAs (miR-21, -29a-3p, -34a, -146a, 148a, -155, -181a, -200a/b, and -224) regulation in TNBC^11,45,57–65^.

Apigenin is a dietary flavonoid, and its role in the inhibition of cancer cell proliferation, invasion, and metastasis has been widely studied in breast, prostate, colon, stomach, cervical, pancreatic, oral, skin, lung, ovarian, and brain cancer using in-vitro and in-vivo studies^66–70^. The chemo-preventive role of Apigenin has been well elucidated in breast cancer using in-vitro and animal studies^71–73^. The transcriptomic analysis showed the anti-cancer and anti-inflammatory effects of Apigenin in TNBC cells by reducing the expression of TNFα, Cathepsin S, and laminin subunit gamma-2^74^. Apigenin inhibited YAP/TAZ activity, suppressed stem cell-like properties, and induced ERβ-mediated cell death in MDA-MB-231 cells^75,76^. The combination of Apigenin with doxorubicin and docetaxel inhibited the activity of P-glycoprotein and BCRP in a synergetic way in multi-drug resistant MDA-MB-231-pcDNA and MDA-MB-231-BCRP cells^77^. In addition, the combination of Apigenin and 5-fluorouracil downregulated the expression of ErbB2, AKT, and AKT proteins, thus inducing apoptotic-mediated cell death in MDA-MB-453^78^. This synergistic combination increased caspase-3 expression and inhibited cell growth by decreasing the resistance of breast cancer cells towards 5-Fluorouracil^78^.

Extensive studies were performed to explore the anti-cancer potential of Apigenin by considering different molecular targets. However, the role of Apigenin in epigenetic modulation and its correlation with micro-RNA regulations in MDA-MB-231 cells is poorly understood. Few reports explained the anti-cancer role of Apigenin through miRNA regulations in various human cancers, but its role in TNBC has remained elusive^79–89^. Hence, this study has been performed to understand the role of Apigenin and its combination with standard HDAC inhibitor (Vorinostat) in modulations of epigenetic regulators, apoptotic markers, and related miRNAs in MDA-MB-231 cells. Finally, in-depth molecular docking and MD simulation studies supported the *in-vitro* results.

## Materials and methods

### In-vitro cell culture studies

The animal cell culture media (Dulbecco's Modified Eagle Medium with high glucose, DMEM) was purchased from Gibco. The 3-(4,5-Dimethylthiazol-2-yl)-2,5-diphenyltetrazolium bromide (MTT) was purchased from Himedia, and 5-(and-6)-chloromethyl-2′,7′-dichlorofluorescein diacetate acetyl ester (CM-H_2_ DCFDA) was procured from Sigma-Aldrich. Annexin V-FITC apoptosis kit was obtained from Bio legend. The apoptosis-specific antibodies against PARP (Cat no-9542T), Bax (Cat no-sc-70405), Bak (Cat no-556382,BD Bioscience), Bid (Cat no-sc-56025), Bcl2 (sc-7382), Caspase-9 (Cat no- sc-133109), HDAC-1(Cat no-34589S), HDAC-3(Cat no-85057S) isoform antibodies and actin (sc-47778) were purchased, antibodies HRP-conjugated secondary mouse (Cat no-sc-2357)and rabbit (Cat no-sc-2768) antibodies were purchased from Santacruz Biotechnology.

### Apigenin and vorinostat (SAHA)

Apigenin (Cat no-A3145**)** and a standard HDAC inhibitor (Vorinostat, SAHA (Cat No- SML0061) were purchased from Sigma-Aldrich. The stock solution was prepared in sterile-filtered dimethyl sulfoxide (DMSO) (Cat no-TC185, Himedia) and stored at − 20 °C.

### Culture and maintenance of MDA-MB-231 cell line

The human origin triple-negative MDA-MB-231 breast cancer cell line was obtained from the National Centre for Cell Science (NCCS), Pune (MS), India a national facility for providing animal cell lines. The cell line was cultured in DMEM (Cat no-11965092, Thermo Fischer) medium supplemented with 10% FBS (Cat no-10270106, Thermo Fischer), penicillin–streptomycin (50 unit/mL; Invitrogen), and was maintained at 5% CO_2_ and 37 °C.

### Elucidating anti-TNBC potential of Apigenin using MTT assay

The anti-TNBC potential of Apigenin alone and in combination with SAHA was assessed by MTT cell proliferation assay using TNBC cancer cells. MTT assay protocol has been used from previous studies^90–94^. In brief, MDA-MB-231 cells were seeded at a cell density of 1 × 10^4^ cells/per well into a 96-well culture plate. The effect of Apigenin and in combination with SAHA on the cell proliferation of MDA-MB 231 cells were tested at concentrations ranging from (10–70 µM) for 48 h. After 48 h the cell culture medium was removed and replaced with 100 μl of MTT reagent (Cat no-TC191, Himedia) [3-(4,5-dimethylthiazol-2-yl)-2,5-diphenyltetrazolium bromide] and incubated at 37 °C for 4 h. The metabolically active cells reduced the yellow tetrazolium dye into an insoluble formazan crystal. Later these crystals were solubilized by adding 100 µL of DMSO, turning the solvent into a purple colour. The formazan product was measured using a Hidex Sense multimode plate reader at 570 nm. The data is represented as the concentration of drugs versus the percentage of cell proliferation. To calculate the IC_50_ values, series of concentrations of Apigenin (10–60 µM) and SAHA (2–10 µM) were tested against the MD-MBA-231 cells and the IC_50_ values were calculated using GraphPad Prism 5.0. For calculation of combination index (CI) of Apigenin with SAHA, serial dilution of their IC_50_ concentrations were prepared and CI was calculated using the Chou Talalay equation^95^.

### Cell migration assay using scratch wound healing

The migration inhibition assay was performed to assess the wound closure effect of Apigenin and its combination with SAHA on MDA-MB-231 cells. The wound healing assay was performed as described in our earlier reports^90–94^. Briefly, the MBA-MD-231 cells were seeded in 24 well plates (1 × 10^4^ cells/well). Sterile pipette tips were used to make scratches in all wells treated with Apigenin, SAHA and their combinations for 48 h. The microscopic images of wound healing of Control, Apigenin, SAHA and their combination were captured at 0 h, 24 h, and 48 h using a phase contrast microscope. Zen software was used to measure the distance of wound healing. The graphs were plotted for time versus distance of wound healing for control and treated cells.

### Reactive oxygen species generation by Apigenin and its combination with SAHA

The role of Apigenin and its combination with SAHA in the generation of intracellular reactive oxygen species (ROS) was investigated by performing a DCFDA assay using the protocol described in our previous study^90,91^. In brief, the MDA-MB-231 cells were seeded in 96 well plates (1 × 10^4^). The cells were treated with Apigenin, SAHA and combination for 48 h. After treatment, the cells were washed with PBS and DCFHDA (Cat no-D6883, Sigma Aldrich) (10 μM) was probed for 20 min followed by washing with PBS. The generation of ROS was measured using a Hidex Sense multimode spectrophotometer and images for the uptake of 2′,-7′-dichlorofluorescein (DCF) were captured using live cell imaging (Zeiss Cell Discoverer 7.0).

### Nuclear staining by DAPI on Apigenin and its combination with SAHA-treated cells

MDA-MB 231 cells were seeded in a 96-well plate at a density of 1 × 10^4^ cells. The cells were treated with flavonoid Apigenin, SAHA and its combination with Apigenin. After 48 h of treatment cells were washed with PBS and incubated with DAPI (Cat No-D1306, Invitrogen) (5 µg/ml) for 20 min. After staining cells were washed once again and visualised for morphology change using a live cell imager (Zeiss Cell Discoverer 7.0).

### Cell cycle analysis using fluorescence-activated cell sorting (FACS)

To investigate the phase distribution of the cell cycle and its regulation after treatment of Apigenin, SAHA and their combination in MDA-MB-231 cells, we performed propidium iodide (PI) staining using flow cytometry analysis^90–94^. The MDA-MB-231 (1 × 10^5^) cells were seeded in 6 well plates and treated with Apigenin, SAHA and their combination. After treatment, cells were stained with Propidium Iodide (Cat no-P4170, Sigma Aldrich) (50 μg/mL). The phase distribution of the cell cycle and its regulation was determined using Flow cytometry. The BD FACSdiva software was used to analyse the results.

### Induction of apoptosis in TNBC cells by Apigenin and its combination with SAHA

To investigate the role of Apigenin, SAHA and their combination in the induction of apoptosis in TNBC cells, we have performed the Annexin-V-FITC assay using Flow cytometry^90–94^. The Annexin V-FITC Apoptosis Detection Kit (Cat no-640906, Bio legend) was used. In brief, the cells were seeded at a density of 1 × 10^5^ in 6 well plates and treated with Apigenin and SAHA and their combination. After 48 h, the cells were washed with PBS (1 ×) and fixed using 70% chilled methanol at − 20 °C for 2 h. Then, the Annexin V-FITC (50 μg/mL) was added followed by PI (50 μg/mL) in control and treated cells. The DNA content of stained nuclei was captured using a flow cytometer (BD Bioscience) and analysis of results was carried out using BD FACSdiva software.

### Quantitative expression of epigenetic regulators (HDACs) using qRT-PCR

The effect of Apigenin, SAHA and their combination on the expression pattern of epigenetic regulators and apoptotic markers was performed using qRT-PCR. The protocol for measuring the expression of mRNA was carried out as per the earlier reported method^90,92,93^. In brief, total RNA from control and treated samples were isolated using TRIZOL reagent (Cat no-15596018, Ambion). The RNA samples were reconstituted in sterile nuclease-free water and quantified using nanodrop (Implen). The cDNA reverse transcription kit (Cat no-1708890, iScript, Bio-Rad) was used for the synthesis of cDNA as per the manufacturer's protocol. The temperature profile was 25 °C for 5 min, 46 °C for 20 min, and 95 °C for 5 min for the reverse transcription using My cycler Thermal Cycler, Bio-Rad. The synthesized cDNA was used for real-time PCR using SYBR green (Cat no-171–5121, Bio-Rad) (CFX96 Real-time System, Bio-Rad) along with epigenetic (HDACs, HAT, DNMT), Pro-apoptotic markers (p53, Caspase-3, Caspase-8, Bax and Bid), anti-apoptotic marker (Bcl2) specific primers and GAPDH-primer as an internal control (Table S1: Supplementary Information). The GAPDH housekeeping gene was used for the data normalization. Fold changes in the mRNA expression levels of epigenetic regulators were analysed using the 2^-ΔΔCT^ method^96^.

### Effect of Apigenin and its combination with SAHA on expression of onco-miRNA and tumour-suppressor miRNAs in TNBC

The MDA-MB-231 cells were grown to 70% confluency and treated with Apigenin at IC_50_ concentration for 48 h. Total micro-RNAs were collected using the Relia Prep miRNA cell and tissue miniprep kit (Cat no-Z6211, Promega), as per the manufacturer’s instructions. The primers for the selected miRNAs were designed using online bioinformatics tools. The real-time RT-PCR (qRT-PCR) was performed as described in the previous study^90,92,93^. Total mRNA was converted to complementary DNA (cDNA) using the miRNA-specific primers for miR-21 and miR-200b (Table S1: Supplementary Information). The miRNA level was amplified using the CFX96 Real-time System, and Bio-Rad PCR System. The PCR conditions consisted of 40 cycles of 95 °C for 5 min, 95 °C for 0.30 s, 60 °C for 30 s, 65 °C for 0.45 s and 95 °C for 0.5 s. The Ct (threshold cycle) value of each primer was normalized to that of RNU6B for miRNA as an internal control.

### Expression profile of epigenetic and apoptosis regulators using immunoblotting analysis

To elucidate the effect of Apigenin, SAHA and their combination on the expression profile of epigenetic regulators (HDACs), pro-apoptotic (Bax, Bak, Bid, Caspase-9, PARP) and anti-apoptotic Bcl2 and Nrf2 markers, we have performed immunoblotting of treated and control sets of MDA-MB-231 cells. The WBA was carried out as per the previously described method^90,92–94^. In brief, the cells at a density of (2 × 10^5^) were seeded in 60 mm plates and treated with Apigenin, SAHA and their combination. After the treatment, the cells were washed with PBS, scraped, pelleted, and lysed in RIPA buffer (Cat no-89900, Thermofisher) containing a protease inhibitor cocktail (Cat no-ML051, Himedia). After incubation for 20 min on ice, the cell lysates were centrifuged at 12,000 rpm for 30 min at 4 °C. The supernatants were collected and protein concentrations were estimated using the Bradford reagent (Cat no-ML106, Himedia). The total cell proteins (30 μg/mL) were electrophoresed on 7.5–12% SDS-PAGE gel using a (Cat no-161-0393, Bio-Rad-All blue protein ladder). After the resolution of proteins, the gels were transferred onto a polyvinylidene fluoride (PVDF) (Cat no-10600021, Cytiva) membrane. The membranes were blocked using skimmed milk prepared in Tris-buffered saline containing 0.1% Tween-20 (TBST) for 1 h. Followed by three consecutive TBST washes the membranes were incubated with an optimal dilution of the desired primary monoclonal antibodies at 4 °C overnight. The membrane was washed three times with TBST and incubated with appropriate secondary antibodies conjugated with horseradish peroxidase (HRP) for 2 h at room temperature. The membranes were exposed to ECL (Cat no- K-12045-D20) Advanta’s detection reagent and the specific protein band was digitalized using the system Amersham Gel-imager-680.

### HDACs inhibition assay

The HDAC inhibition potential of Apigenin and its combination was tested using an HDAC inhibition kit. The MDA-MB-231 cells were harvested at the indicated time points and the nuclear extract was prepared using a nuclear extraction kit (EpiQuik™ Nuclear Extraction Kit Cat. No: OP-0002-1). The HDACs inhibition by Apigenin, SAHA and their combinations was determined using total nuclear extract obtained from MDA-MB-231 cells as an HDAC source and the enzymatic HDAC activity measurement was performed using a fluorometric HDAC assay kit (Epigentek, Base Catalog # P-4034) according to the manufacturer’s instructions (https://www.epigentek.com/docs/P-4034.pdf). Briefly, the cells were washed twice with ice-cold PBS and the pellet was collected by centrifugation. The nuclear extract treated with apigenin, SAHA and their combinations were incubated with various concentrations for 1 h at 37 °C in the presence of an HDAC fluorometric substrate. The fluorescence was measured after adding the HDAC assay developing solution and within 5–10 min of incubation at room temperature using a spectrofluorometer with excitation at 450 nm and 650 nm. The measured activities were calculated using HDAC Activity (OD/min/mg) = (Sample OD – Blank OD)/ (Protein Amount (µg) * × min**) × 1000.

### HAT activity assay

The effect of Apigenin and its combination in the regulation of HAT activity was accessed by performing the HAT inhibition kit. The MDA-MB-231 cells were harvested at the indicated time points and the nuclear extract was prepared using a nuclear extraction kit (EpiQuik™ Nuclear Extraction Kit Cat. No.: OP-0002-1). The EpiQuik-HAT-Activity/Inhibition Assay Kit (Catalogue # P-4003) was used to determine HAT activity. The manufacturer’s protocol was followed for the preparation of nuclear extract (EpiQuik Nuclear extraction kit I; Catalogue number #OP-002) and determining HAT activity (EpiQuik HAT Activity/Inhibition Assay Kit; Catalogue #P-4003). Overall, HAT activity was calculated using HAT activity (OD/h/mg protein) = OD (untreated sample – blank) × 1000/h × protein amount (µg) added into the assay.

### DNMTs inhibition assay

The cultured MDA-MB-231 cells were harvested at the indicated time points and the nuclear extract was prepared with the nuclear extraction reagent. The DNMT activity was determined using the EpiQuik DNA methyltransferase activity assay kit (Epigentek, Base Catalog # P-3001) according to the manufacturer's protocol (https://www.epigentek.com/docs/P-3001.pdf) and earlier described method^97^. This analysis has provided the overall DNMT activity and the data were represented in terms of percentage of enzyme inhibition as compared to control.

### Molecular docking of Apigenin and SAHA with HDAC1 and HDAC3

To corroborate our experimental results of qRT-PCR and western blot analysis and to explore the mechanism of catalytic inhibition of HDAC1 and HDAC3 by Apigenin and SAHA, we have performed molecular docking studies. The Genetic Optimization for Ligand Docking (GOLD) program v5.2.2 has used for molecular docking studies^98^. During the docking study, the genetic algorithm provides partial flexibility to receptor proteins and total flexibility to ligand molecules. The crystal structure coordinates for HDAC1 (PDB code: 5ICN) and HDAC3 (PDB code: 4A69) were extracted from the protein data bank (www.rcsb.org) and used for molecular docking^99,100^. The receptor proteins were prepared by removing the water molecules and hydrogen atom addition to receptor structures. The ND1H protonation state has maintained for the histidine tautomer’s present in the active site pocket of HDAC1 and HDAC3. The binding pockets were specified by selecting the area of 10 Å of co-crystallized ligands present in the active-site pockets of HDAC1 and HDAC3. Gold score and Chemscore functions have used to predict the binding affinities between the receptors and ligands. The 100 docking poses were generated for each ligand molecule, and the gold score and Chemscore were used to select the best pose of ligands within the binding pocket of HDAC1 and HDAC3. Molecular interactions such as hydrogen bonding, π-stacking, metal-coordination, and hydrophobic contacts were analysed for the stable docked complexes of Apigenin/SAHA and HDAC1/3. The final docked complexes of Apigenin and SAHA with HDAC1/3 have subjected to molecular dynamics simulation studies.

### Molecular dynamics (MD) simulation

The MD simulations of 100 ns were performed on stable docked complexes of Apigenin and SAHA with HDAC1/3 using the GROMACS 2018 package with a Gromos96 force field^101,102^. The topology files of Apigenin and SAHA were generated using PRODRUG online webserver^103^. The TIP3P water model was used to solvate the HDAC1 and HDAC3 system by applying 10 Å of edged cubic box. The Na^+^ counter ions were added to neutralize the system. To remove unfavourable contacts from initial structures, 10,000 steps of energy minimization were performed using steepest descent method until the tolerance of 2000 kJ/mol was achieved. After energy minimization three steps of equilibration were performed. At the first phase of equilibration, the Nose–Hoover thermostat was used to maintain a constant temperature at 300 K for 100 ps^104^. Later, a 100 ps NPT ensemble was applied at 1 bar of pressure followed by 100 ns of the production run under the same ensembles. The Parrinello-Rahman barostat method was used to maintain the pressure of the system during simulations^105^. The protein backbone of HDAC1 and HDAC3 were restrained and solvent molecules with counter ions were allowed to move during the equilibration process. The LINCS algorithm was applied to restrain the all bonds to a hydrogen atom using a 2 fs of time step^106^. The long-range electrostatic interactions were calculated by employing the particle mesh Ewald (PME)^107^. The cut-off distances of 9 Å and 10 Å were used to calculate the Coulombic and van der Waals interactions, respectively. The MD simulations were performed by releasing all constraints along with the periodic boundary conditions to avoid edge effects^108^. The 2 fs of time step was used throughout the simulation and the coordinate data of trajectories were stored at every picosecond (ps). The simulation results were analysed using GROMACS, VMD, and Discovery studio.

### Binding-free energy calculation by using MM-PBSA

The binding free energy of protein–ligand complexes of Apigenin and SAHA with HDAC1 and HDAC3 were calculated using the Molecular Mechanics Poisson-Boltzmann Surface Area (MM-PBSA) method^109^. The average binding energy was calculated by analysing 20 snapshot structures of each simulated complex from the last 20 ns of MD trajectories. The g_mmpbsa tool of GROMACS was employed to calculate the contribution of different energetic parameters such as van der Waals (ΔE_vdw_), electrostatic (ΔE_elec_), non-polar solvation (ΔG_nps_), and polar solvation (ΔG_Ps_) energy in the total binding energy. Also, the residual contribution of key residues in binding free energy was calculated by the MmPbSaDecomp.py Python script.

Binding energy was calculated as;

$$ \begin{gathered} \Delta {\text{G}}_{{{\text{bind}}}} = \, \Delta {\text{E}}_{{{\text{MM}}}} + \, \Delta {\text{G}}_{{{\text{Solv}}}} \hfill \\ \Delta {\text{E}}_{{{\text{MM}}}} = \, \Delta {\text{E}}_{{{\text{vdw}}}} + \, \Delta {\text{E}}_{{{\text{elec}}}} \hfill \\ \Delta {\text{G}}_{{{\text{Solv}}}} = \, \Delta {\text{G}}_{{{\text{nps}}}} + \, \Delta {\text{G}}_{{{\text{ps}}}} \hfill \\ \end{gathered} $$

### Statistical analysis

All the experiments were done in triplicates. The statistical analysis was done by GraphPad-PRISM version 5.01 using one-way ANOVA. The error bar represents mean ± SD derived from three independent replicates. The *, ** and *** denotes p-values ≤ 0.05, ≤ 0.01 and ≤ 0.001, respectively.

## Results

### Effect of Apigenin and its combination with SAHA on cell viability of MDA-MB-231 cells

**Figure 1:**
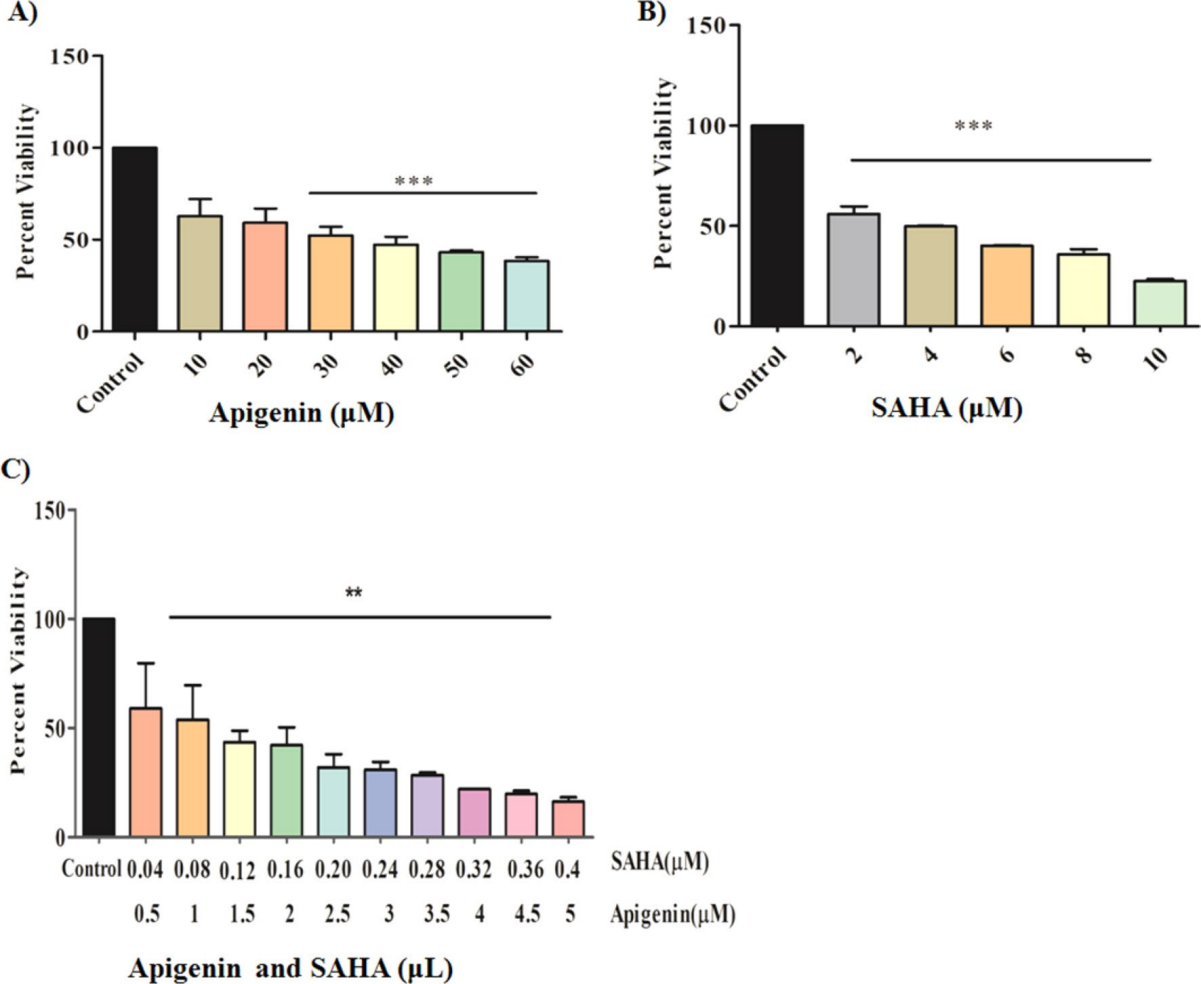
Effect of (**A**) Apigenin, (**B**) SAHA, and (**C**) combination of Apigenin and SAHA on cell viability of MDA-MB-231 cells.

The effect of Apigenin, SAHA, and their combinations on the cell viability of triple-negative breast cancer (MDA-MB-231) cells was studied by using the MTT assay. Figure 1 depicts the dose-dependent inhibition of cell proliferation of MDA-MB-231 cells. The decrease in cell viability of MDA-MB-231 cells was observed at the increasing concentrations of Apigenin, SAHA, and their combinations (Fig. 1A–C).

**Figure 2:**
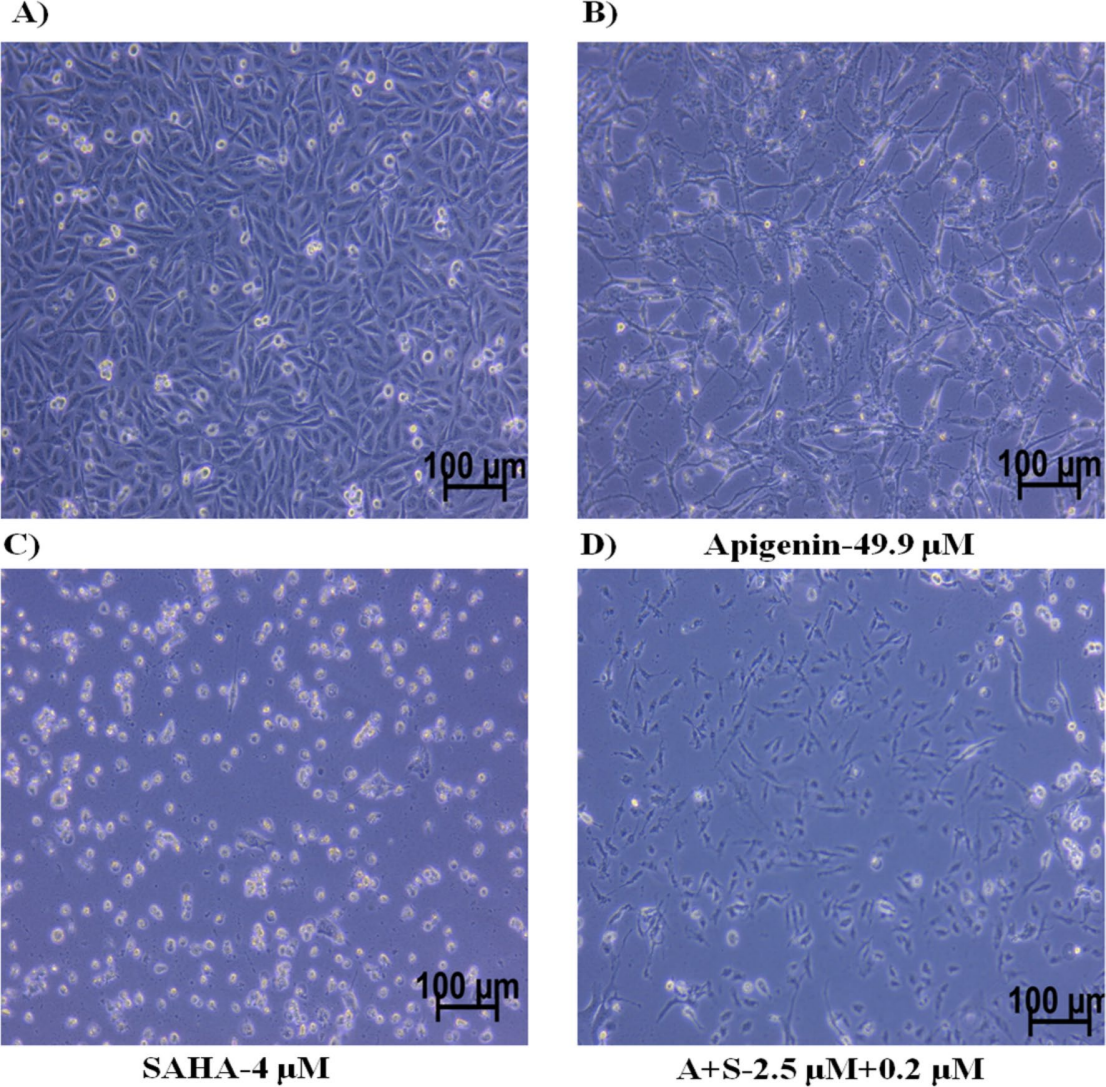
Morphological changes induced by flavonoids in MDA-MB-231 cells; (**A**) control, (**B**) Apigenin-treated, (**C**) SAHA-treated, (**D**) combination of Apigenin and SAHA-treated cells.

The nearly 50% growth inhibition of MDA-MB-231 cells was noticed at IC_50_ = 49.90 µM/mL (equivalent to 13.48 μg/mL) of Apigenin. The MDA-MB-231 cells are more sensitive to the reference drug ‘SAHA’, which displayed 50% of cell death at IC_50_ = 4 µM/mL (0.2 μg/mL) while 90% of cell death was observed at 10 µM/mL concentration (Fig. 1B). The calculated combinatorial index values for SAHA and Apigenin are 0.2 µM/mL and 2.5 µM/mL, respectively (Fig. 1C). At this concentration, 50% of the cell death was observed in MDA-MB-231 cells. In the combinatorial study, SAHA and Apigenin have improved their anti-TNBC potential synergistically and showed good inhibitory activity against MDA-MB-231 cells. As compared to control cells, the Apigenin, SAHA, and their combinations induced adverse morphological changes in TNBC cells (Fig. 2). This suggested that Apigenin works effectively in combination with SAHA against TNBC cells as compared to its inhibitory effect at the individual level.

### Apigenin and its combination with SAHA demonstrated an enhanced ability to inhibit the cell migration of MDA-MB-231 cells

**Figure 3:**
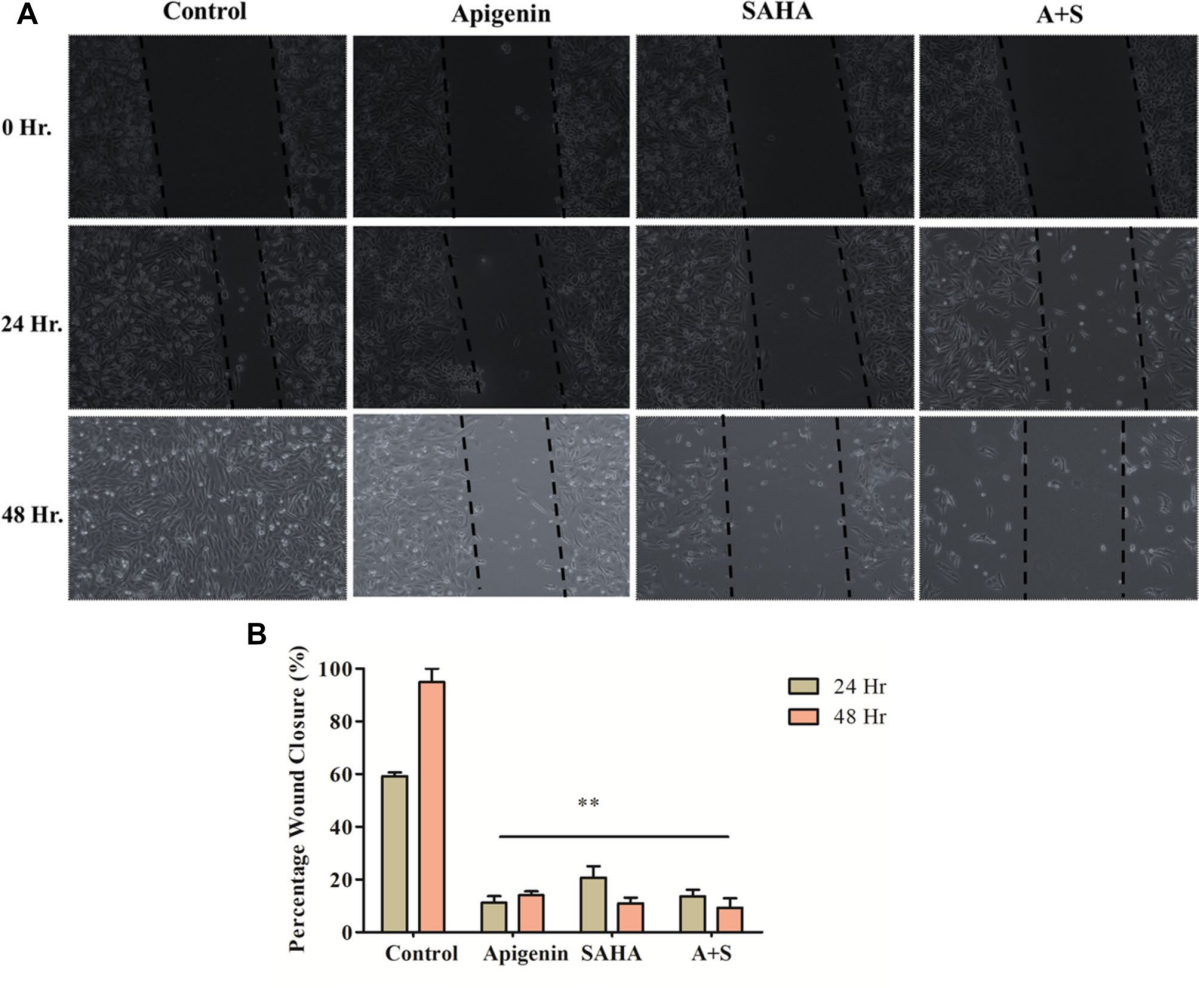
(**A**) Digitized images showing the effect of Apigenin, SAHA, and their combinations on wound closure ability of MDA-MB-231 cells after 24 h and 48 h. (**B**) Percentage of wound closure after the treatment of Apigenin, SAHA, and their combinations in MDA-MB-231 cells after 24 h and 48 h.

The effect of bioactive anticancer agents on the cell migration ability of cancer cells has been commonly investigated by using the wound healing assay. The Apigenin, SAHA, and their combination showed promising anti-migration and thereby anti-metastatic potential against TNBC by inhibiting the migration potential of MDA-MB-231 cells (Fig. 3A). The normal pattern of cell migration as well as wound healing was observed in control cells (Fig. 3A). In contrast, 90–95% of the wound healing/migration ability of MDA-MB-231 cells was inhibited after the treatment of Apigenin for 24 h and 48 h (Fig. 3B). SAHA reduced the cell migration ability of MDA-MB-231 cells by 85–90%. Similar results were observed in the combinatorial study of Apigenin and SAHA, focusing on the significance of combinatorial efficacy (Fig. 3B).

Apigenin alone and in combination with SAHA inhibited the cell migration/wound healing ability and metastatic potential of MDA-MB-231 cells. Based on these results, we anticipated that the mode of action of Apigenin might be the same as that of reference drug ‘SAHA,’ and may act as a complementary anticancer agent by decreasing the metastasis and invasive potentials of MDA-MB-231 cells.

### Apigenin and its combination with SAHA generates reactive oxygen species in MDA-MB-231 cells

**Figure 4:**
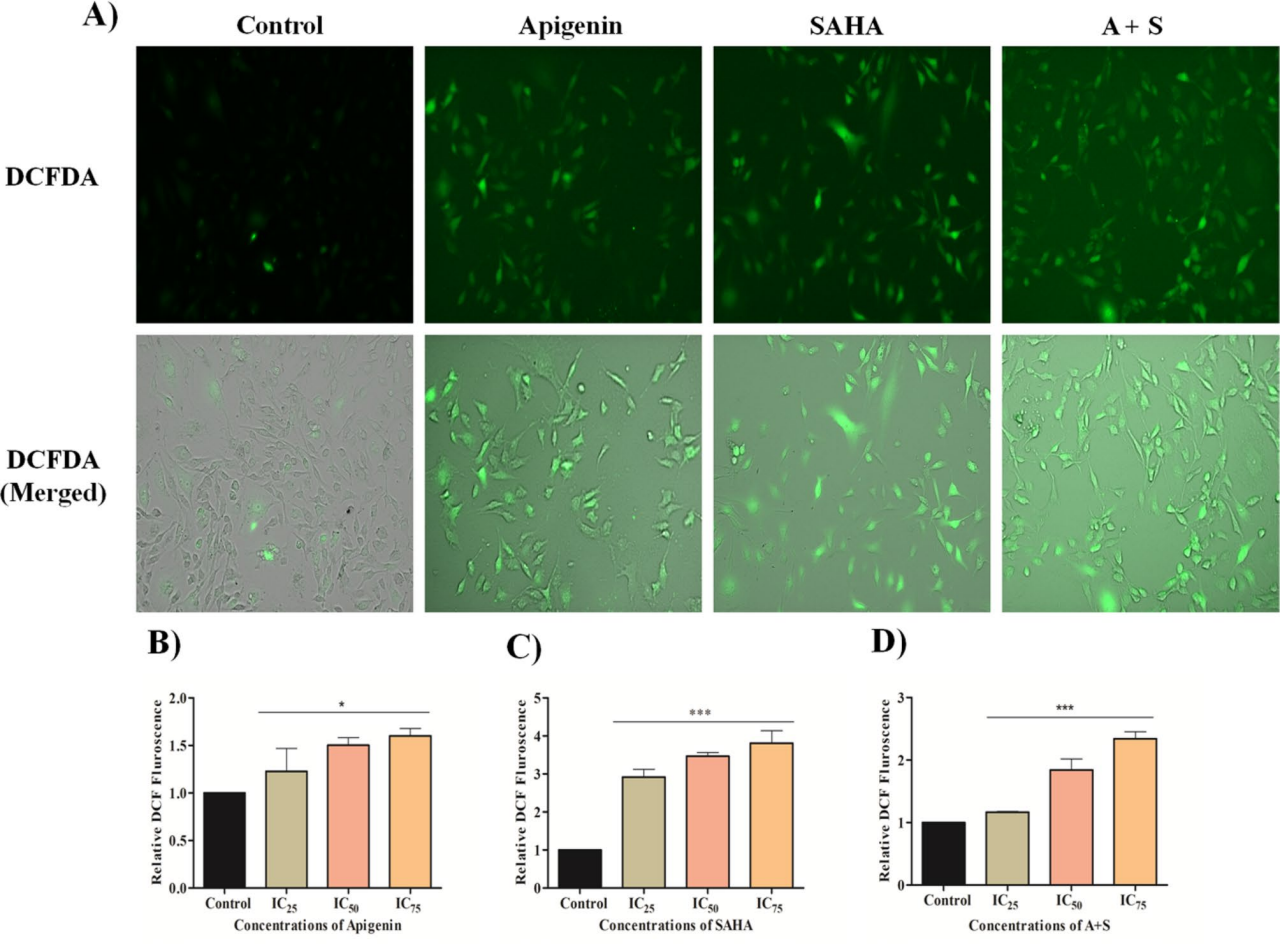
ROS generation in MDA-MB-231 cells after 48 h of treatment of Apigenin, SAHA, and a combination of Apigenin and SAHA. The relative DCF fluorescence after the treatment of (**B**) Apigenin, (**C**) SAHA, and (**D**) Combination of Apigenin and SAHA in the induction of ROS generation in MDA-MB-231 cells.

The DCFDA staining was performed using a colorimetric assay and live cell imaging studies to elucidate the role of Apigenin, SAHA, and their combinations on the generation of intracellular reactive oxygen species (ROS) in MDA-MB-231 cells (Fig. 4). The high uptake of DCFDA was observed in Apigenin-treated cells which signify that MDA-MB-231 cells generated a high level of ROS as compared to control cells (Fig. 4A). At increasing concentrations of Apigenin, a more ROS generation was observed in TNBC cells (Fig. 4B).

More cell death was observed at higher uptake of DCFDA by Apigenin-treated TNBC cells. Similarly, SAHA and a combination of Apigenin with SAHA were induced high ROS generation in TNBC cells (Fig. 4C,D). This suggests that Apigenin may act as a potent anti-TNBC agent like SAHA by inducing apoptotic-mediated cell death.

### Apigenin and SAHA-induced nuclear fragmentation and chromatin condensation in TNBC cells

**Figure 5:**
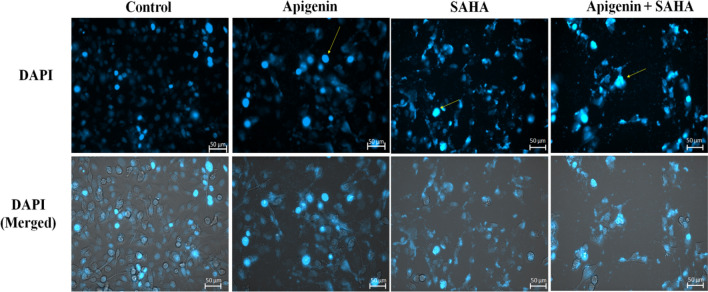
Apoptotic cell morphology of the MDA-MB-231 cells after treatment of Apigenin, SAHA, and their combinations detected by fluorescent live cell microscopy after DAPI staining.

The Apigenin, SAHA, and their combined treatment induced nuclear fragmentation and chromatin condensation in MDA-MB-231 cells (Fig. 5). The reduction in cell division of MDA-MB-231 cells was observed after the treatment of Apigenin, SAHA, and their combinations as compared to control cells. This indicates that Apigenin efficiently induces apoptotic-mediated cell death in TNBC cells.

### Apigenin reduced mitochondrial membrane potential (ΔΨM) in MDA-MB-231 cells

**Figure 6:**
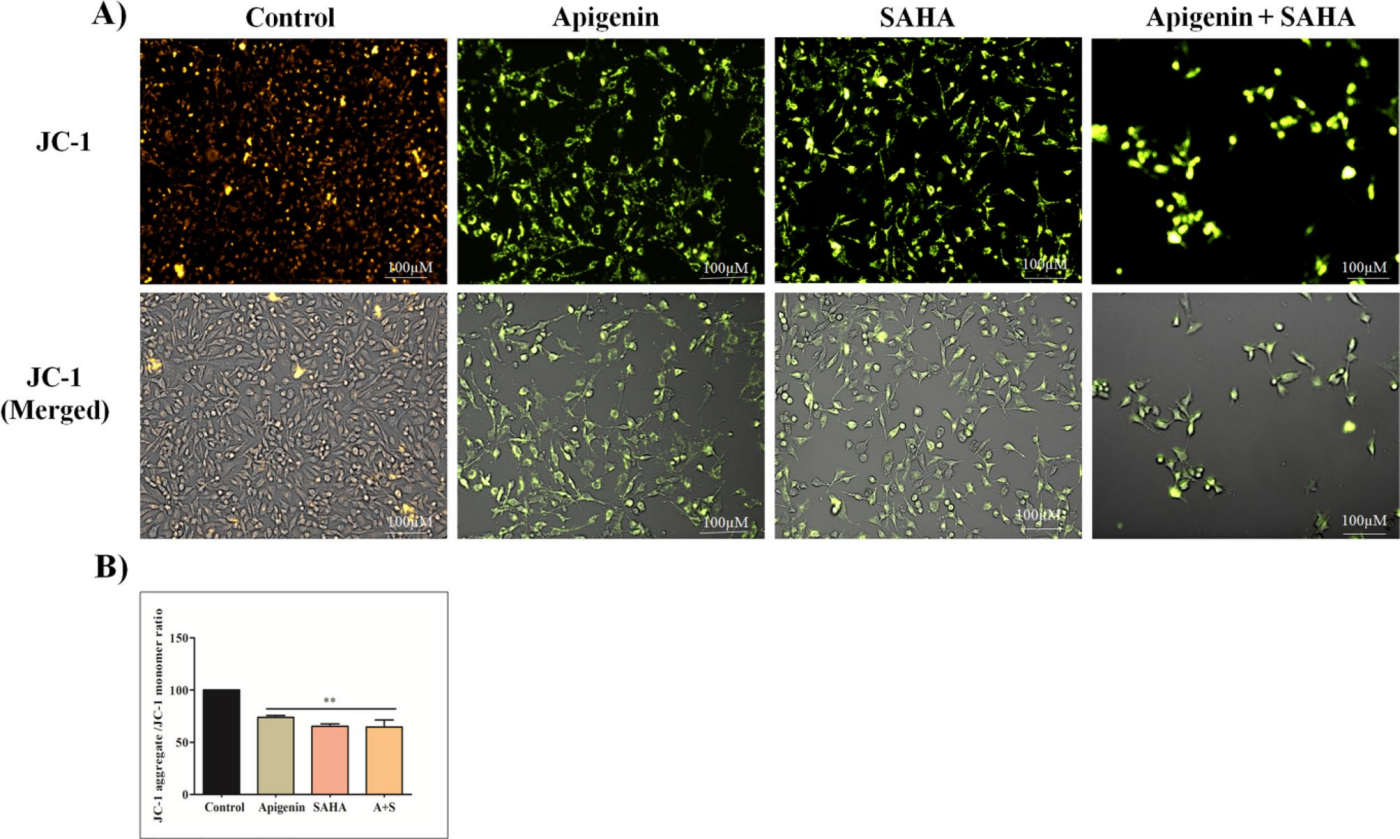
(**A**) Effect of Apigenin, SAHA, and their combinations on mitochondrial membrane potential in MDA MB-231 cells after treatment of 48 h. Red fluorescence shows JC-1 aggregates accumulated in mitochondria due to increased ΔΨm, whereas green fluorescence represents monomeric JC-1 in the cytoplasm indicating a decrease in ΔΨm and (**B**) the relative statistical representation of a decrease in ΔΨm after the treatment of Apigenin, SAHA, and AS.

The reduction of mitochondrial membrane potential (ΔΨM) in MDA-MB-231 cells was observed after the treatment of Apigenin, SAHA, and their combination (Fig. 6). The 5,5,6,6’-tetrachloro-1,1’,3,3’ tetraethylbenzimi-dazoylcarbocyanine iodide (JC-1) dye was used to detect ΔΨM in healthy and drug-treated MDA-MB-231 cells. The JC-1 is a lipophilic and cationic dye that enters the cell mitochondria, where it accumulates and starts forming ‘J-aggregates’ reversible complexes in a concentration-dependent manner. These J-aggregates display excitation and emission in the red spectrum (maximum at ~ 590 nm) instead of green. In healthy TNBC cells possessing a normal ΔΨM, the JC-1 dye enters and accumulates in the energized and negatively charged mitochondria and spontaneously forms red fluorescent J-aggregates which are depicted in Fig. 6A.

In contrast, the JC-1 dye also enters the cell mitochondria of Apigenin and SAHA-treated MDA-MB-231 cells but to a lesser extent, since the inside of the mitochondria is less negative because of increased membrane permeability and consequent loss of electrochemical potential.

Under this condition, JC-1 does not reach a sufficient concentration to trigger the formation of J-aggregates, thus retaining its original green fluorescence (Fig. 6). Significant increase in green fluorescence indicates the activation of apoptosis and induction of apoptotic-mediated cell deaths in Apigenin and SAHA treated cells. Similar results were observed in a combinatorial study of Apigenin and SAHA (Fig. 6A,B) The JC-1 observations are consistent with DCFDA results, which suggest the apoptotic-mediated cell death in TNBC cells induced by Apigenin.

### Apigenin, SAHA, and their combination induced cell cycle arrest and apoptotic-mediated cell death in MDA-MB-231 cells

**Figure 7:**
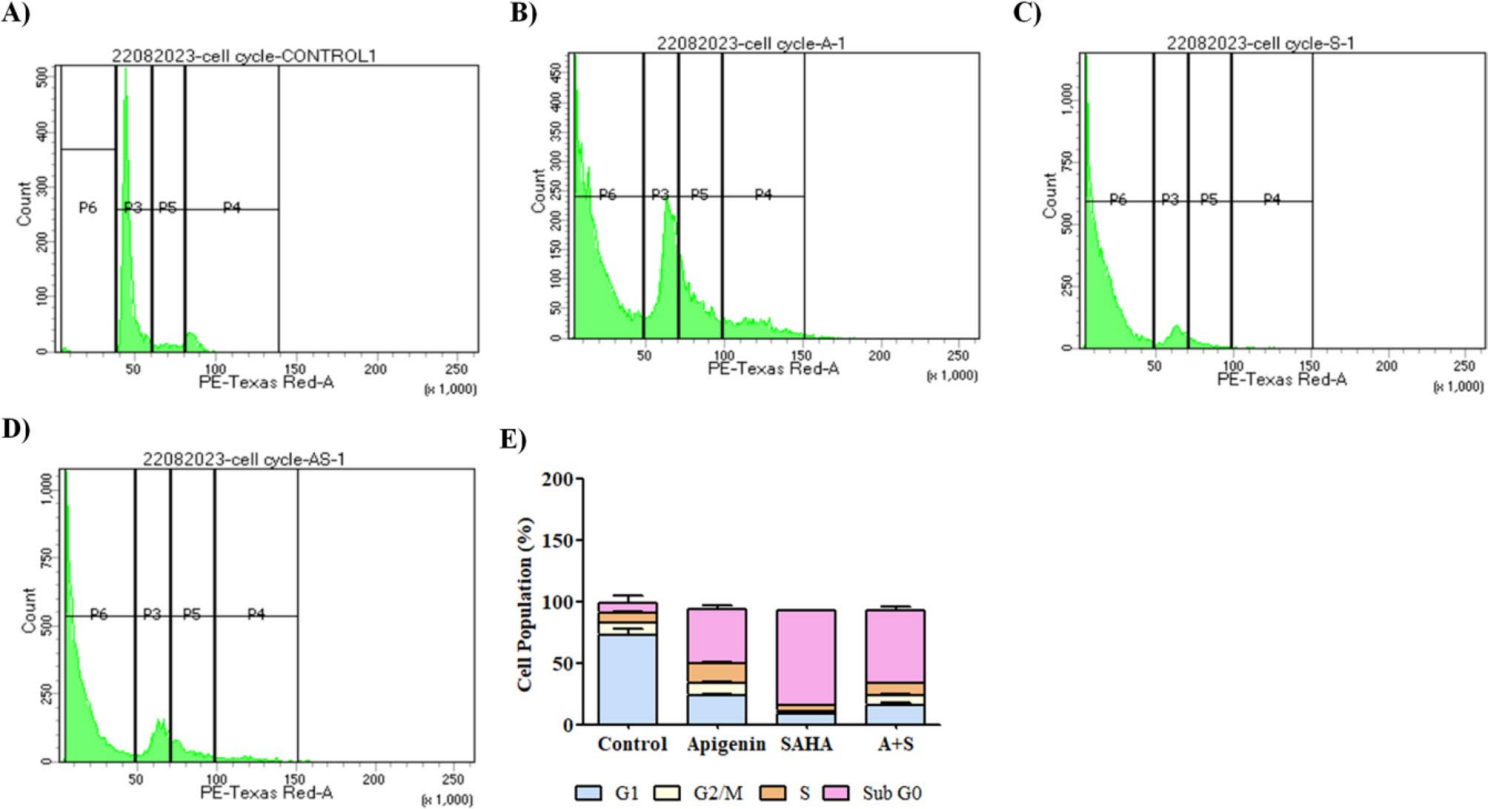
Effect of Apigenin, SAHA and their combinations on the cell cycle regulation in MDA-MB 231 cells (**A**) control cells (without treatment), (**B**) apigenin arrest cells at subG0/G1 phases, (**C**) SAHA arrest cell cycle at subG0/G1 phases, (**D**) combination of Apigenin and SAHA arrest cell cycle at subG0/G1 phases, and (**E**) calculated % of cells population in different phases of the cell cycle.

The effect of Apigenin, SAHA, and their combination on cell cycle regulation of MDA-MB-231 cells was investigated by PI staining using FACS analysis (Fig. 7). In the control sample, more than 86.9% of cells were present in the G1 and S phases, which indicated normal cell cycle regulation (Fig. 7A,E). Whereas, Apigenin arrested 70.7% of cells in the subG0/G1 phases of the cell cycles (Fig. 7B,E). The SAHA arrested 86.6% of cells in subG0/and G1 phases in the MDA-MB-231 cells (Fig. 7C,E). Similar results were noticed in the combinatorial treatment of Apigenin and SAHA, where 77% of cells were arrested in subG0/and G1 phases (Fig. 7D,E). This suggests that Apigenin and SAHA have a similar mode of action against breast cancer cells in the regulation of the cell cycle (Fig. 7).

### Apigenin, SAHA, and their combination induced apoptosis in TNBC cells

**Figure 8:**
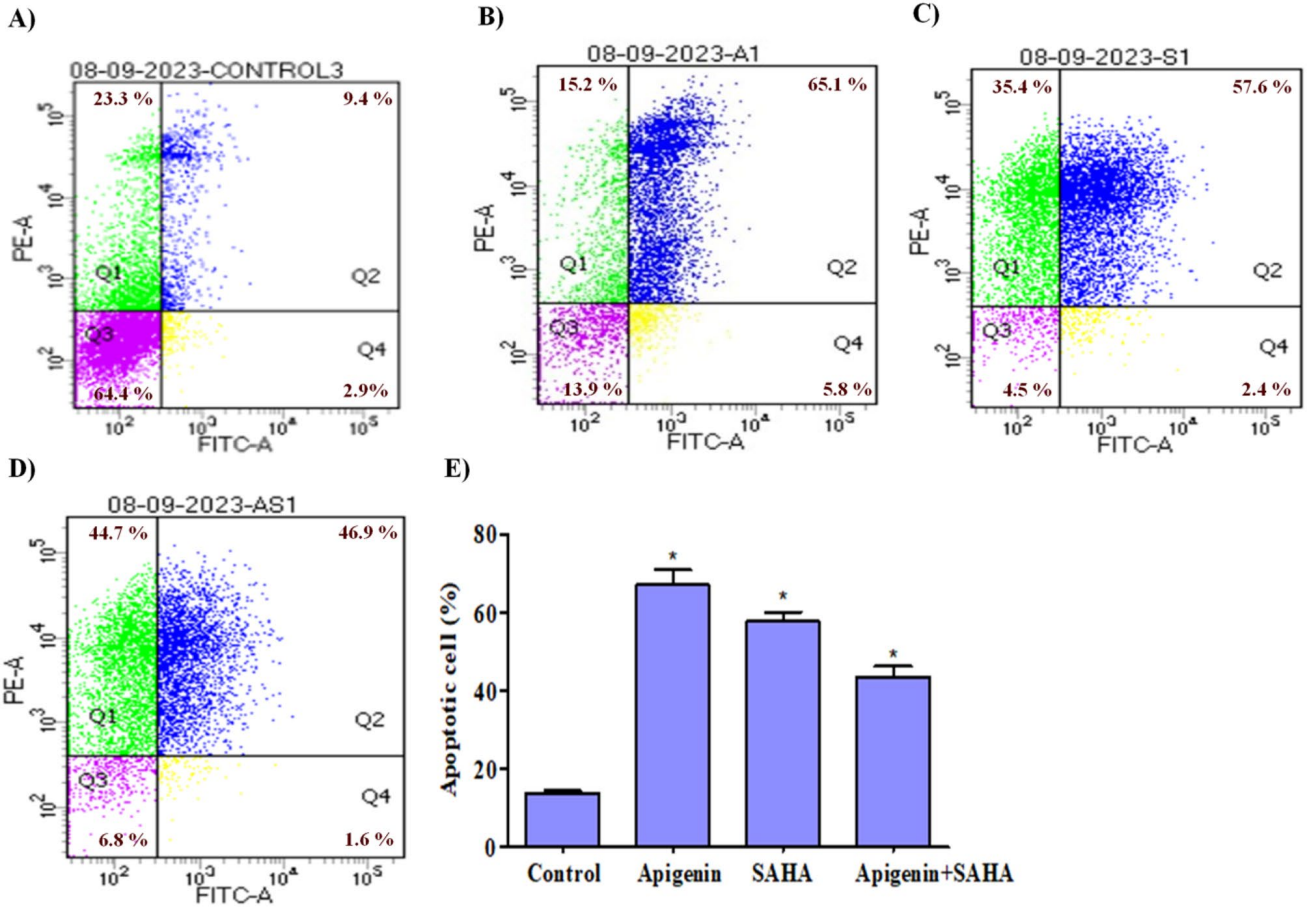
Profile of apoptotic-mediated cell death in MDA-MB-231 cells; (**A**) control cells (without treatment), (**B**) apigenin, (**C**) SAHA, and (**D**) Combination of apigenin and SAHA-induced apoptotic mediated cell death. (**E**) Graph showing the percent of cells arrested in MDA-MB-231 after the treatment of Apigenin, SAHA, and their combination (the bars represent the percentage of cells from Quadrant, Q1: Necrosis, Q2 = Apoptotic, Q3 = Live, and Q4 = Early Apoptotic cells).

In addition to cell cycle analysis, an Annexin V-FITC flow cytometry study was carried out to understand the role of Apigenin, SAHA, and their combination in the induction of apoptotic-mediated cell death in MDA-MB-231 cells (Fig. 8). In the control sample, 64.4% of cells were alive, while Apigenin, SAHA, and a combination of Apigenin with SAHA treated samples had 13.9%, 4.5%, and 6.8% live cells, respectively (Fig. 8B–E). Apigenin induced 70.9%, SAHA 60.0% and a combination of Apigenin and SAHA induced 48.5% of apoptotic-mediated cell death in MDA-MB-231 cells after 48 h of treatment (Fig. 8B–E). However, necrotic cell death was higher in SAHA and a combination of Apigenin and SAHA-treated cells. The outcome of DCFDA, DAPI, JC-1, and FACS analysis indicate that Apigenin and its combination with SAHA induce apoptotic-mediated cell death by generating high ROS, nuclear fragmentation, and chromatin condensation, and arresting the cell cycle at the subG0/G1 phases.

These results are comparable to SAHA, thereby helping to conclude that Apigenin may have a similar mode of action in generating ROS and arresting the cell cycle in MDA-MB-231 cells. Therefore, we anticipate that Apigenin and its combination with SAHA may be a suitable strategy for the treatment of breast cancer.

### Apigenin, SAHA, and their combination modulated the expression of epigenetic regulators (HDACs and DNMT), pro-apoptotic and anti-apoptotic markers in MDA-MB-231 cells

**Figure 9:**
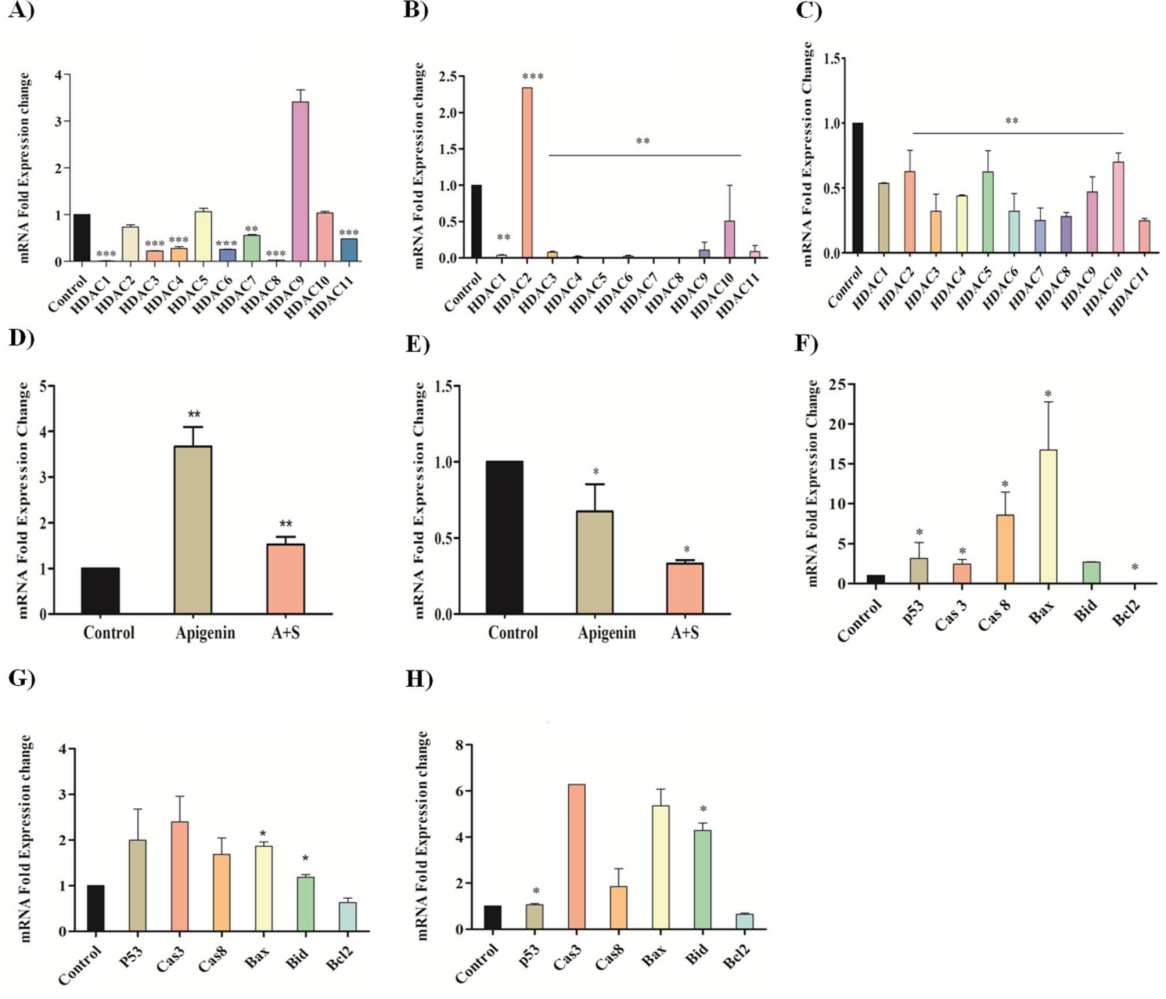
Transcriptomic analysis of effect of Apigenin, SAHA, and their combinations on expression of epigenetic modulators and pro- and anti-apoptotic proteins in MDA-MB-231 cells; (**A**) effect of apigenin treatment on the expression of HDAC isomers and its comparison with (**B**) SAHA, (**C**) Combination of Apigenin and SAHA modulated the expression profile of HDAC isomers, (**D**) Increased the HAT activity, and (**E**) inhibited DNMT expression levels. (**F**) Apigenin regulated expression profile of pro- and anti-apoptotic proteins and its comparison with (**G**) SAHA, (**H**) combined effect of Apigenin and SAHA on the expression of pro- and anti-apoptotic proteins.

The role of Apigenin, SAHA, and their combination in the modulation of expression profiles of epigenetic regulators (HDACs and DNMT), pro-apoptotic (p53, Cas3/8, Bax and Bid) and anti-apoptotic (Bcl2) marker in MDA-MB-231 cells has been elucidated by using qRT-PCR studies (Fig. 9).

In MDA-MB-231 cells, Apigenin, SAHA, and their combination inhibited the expression of class I, II, and IV HDACs (Fig. 9A–C). Apigenin downregulated the expression profiles of HDAC isoforms, except HDAC5/9/10 (Fig. 9A). Remarkably, Apigenin reduced the expression levels by 90–95% of HDAC1 and HDAC8 in TNBC cells. Further, the treatment of Apigenin resulted in a 70–80% reduction in the expression profiles of HDAC3/4/6, while HDAC2, 7, and 11 were downregulated by 30–50% and HDAC9 was observed to be upregulated in MDA-MB-231 cells (Fig. 9A). The profile of Apigenin regulated HDACs expression was compared with SAHA treatment, wherein it was found that all HDAC isomers were downregulated by 90–95%, except HDAC10 which downregulated by 60% as compared to control cells (Fig. 9B). However, the HDAC2 was upregulated after the treatment of the SAHA. Interestingly, the combinatorial treatment of Apigenin with SAHA downregulated the expression of all HDACs in MDA-MB-231 cells (Fig. 9C). In contrast, the expression of HAT was found to be upregulated in MDA-MB-231 cells after the treatment of Apigenin and its combination with SAHA (Fig. 9D). Like HDACs, the expression of DNMT was reduced after the treatment of Apigenin and its combination with SAHA as compared to control cells (Fig. 9E). The overall qRT-PCR results of epigenetic regulators are analogous to each other after the treatment of Apigenin, SAHA and their synergetic combination, and help to maintain the equilibrium of epigenetic regulators in MDA-MB-231 cells (Fig. 9).

In addition, Apigenin, SAHA, and their combination modulated the expression profiles of pro-apoptotic markers (p53, Cas3/8, Bax and Bid) and anti-apoptotic Bcl2 protein in MDA-MB-231 (Fig. 9F–H). Apigenin increased the expression of pro-apoptotic markers including tumour suppressor p53, Caspase-3, Caspase-8, Bax, and Bid while reducing the expression of anti-apoptotic Bcl2 marker in TNBC cells (Fig. 5F). Like Apigenin, the SAHA also increased the expression of tumour suppressor p53 and pro-apoptotic Caspase-3, Capase-8, Bax and Bid markers and downregulated Bcl2 (Fig. 5G). In the combinatorial study, the tumour suppressor p53 was slightly upregulated as compared to the control, while Cas3/8, Bax, and Bid were remarkably upregulated in TNBC cells (Fig. 9H). Here also, the downregulation of the anti-apoptotic Bcl2 marker was observed. Like epigenetic regulators, the proper equilibration between the expression profiles of pro- and anti-apoptotic markers was noticed in MDA-MB-231 cells after the treatment of Apigenin, SAHA, and their synergetic combination (Fig. 9). These observations suggest that the mechanism of modulations of epigenetic regulators, pro- and anti-apoptotic markers by Apigenin is like that of reference drug SAHA.

### Apigenin, SAHA, and their combination modulated the expression of epigenetic regulators and apoptotic markers at the proteomic level

**Figure 10:**
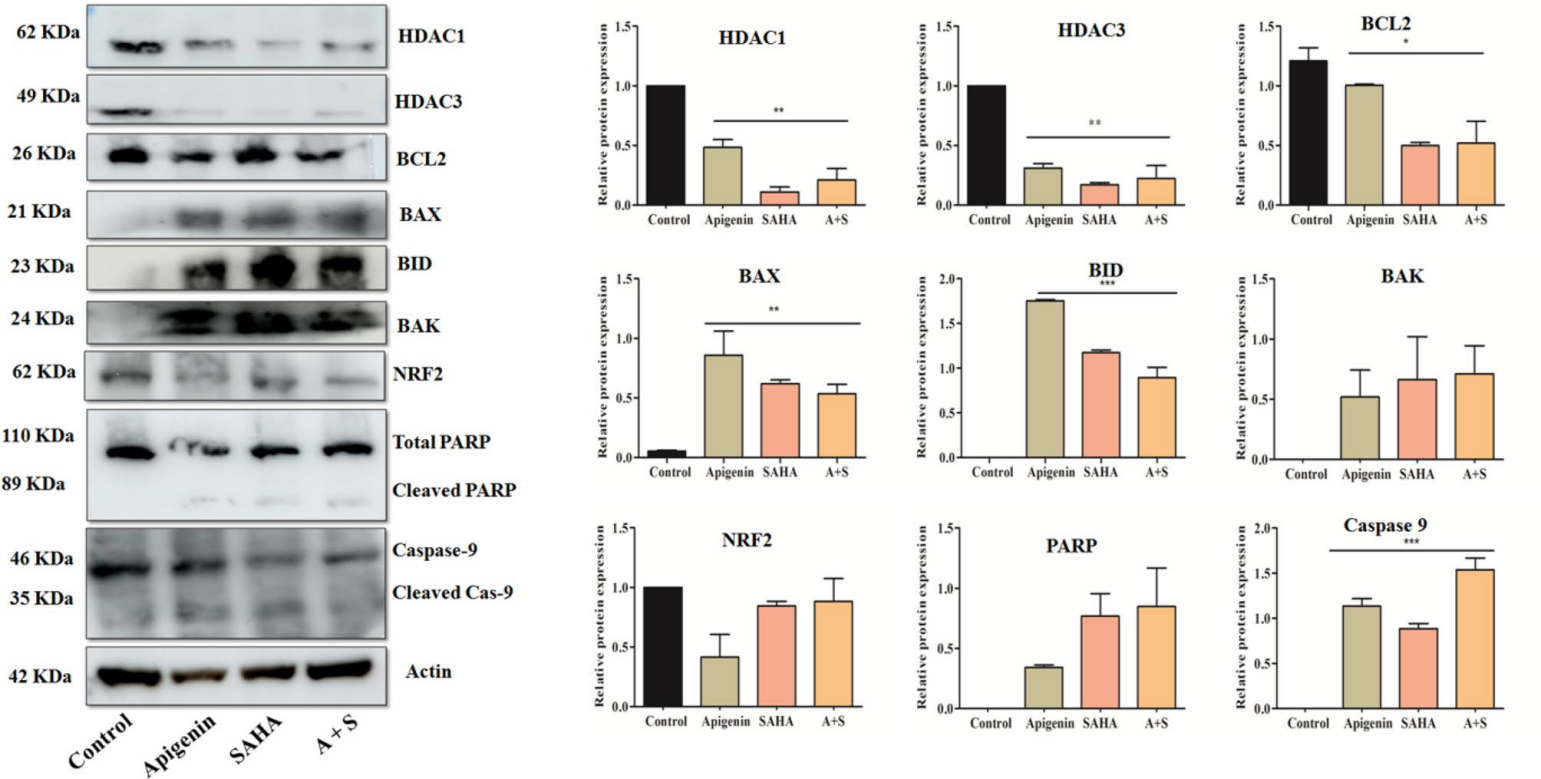
Immunoblotting analysis showing the effect of Apigenin, SAHA, and their combination on expression profiles of epigenetic regulators (HDAC1 and HDAC3), pro-apoptotic (Bax, Bak, Bid, Caspase-9 and Parp) and anti-apoptotic (Bcl2 and Nrf2) protein markers in control and treated MDA-MB 231 cells.

The western blot analysis was performed to elucidate the effect of Apigenin, SAHA, and their combination in the regulation of expression profiles of epigenetic regulators (HDAC1 and HDAC3), pro-apoptotic (Bax, Bak, Bid Caspase9, and PARP) markers, anti-apoptotic protein Bcl2 and transcription factor (Nrf2) at a proteomic level (Fig. 10). Apigenin reduced the expression profiles of HDAC1 and HDAC3 regulators by 0.5 and 0. 7 folds in MDA-MB-231 cells, respectively, as compared to untreated cells (Fig. 10). Whereas, the Bax, Bid, and Bak proteins were upregulated by 0.7, 1.8, and 0.5 folds, respectively in Apigenin-treated TNBC cells as compared to control cells (Fig. 10).

The anti-apoptotic protein Bcl2 and transcription factor (Nrf2) were downregulated by 0.3 and 0.7 folds, respectively as compared to control cells (Fig. 10). Further, Apigenin increased the cleaved products of PARP (0.3-fold) and Caspase-9 (1.2-fold) in MDA-MB-231 cells (Fig. 10). The reduction in expression profiles of HDAC1, HDAC3, and Bcl2 proteins was more in SAHA treated cells as compared to Apigenin but Apigenin produced a significant effect in overexpression of Bax and Bid proteins as compared to SAHA. In contrast, SAHA has less effect in the downregulation of Nrf2, while a noticeable effect in the upregulation of Bak as compared to Apigenin-treated MDA-MB-231 cells. The cleaved product of Caspase-9 was less in SAHA-treated cells as compared to Apigenin-treated cells and *vice-versa* in the case of the cleaved product of Parp. Similarly, a combinatorial study of Apigenin and SAHA maintained the equilibrium between the expression profiles of pro-apoptotic and anti-apoptotic markers in MDA-MB-231 cells (Fig. 10).

### Apigenin demonstrated the inhibition of the enzymatic activity of HDACs

**Figure 11:**
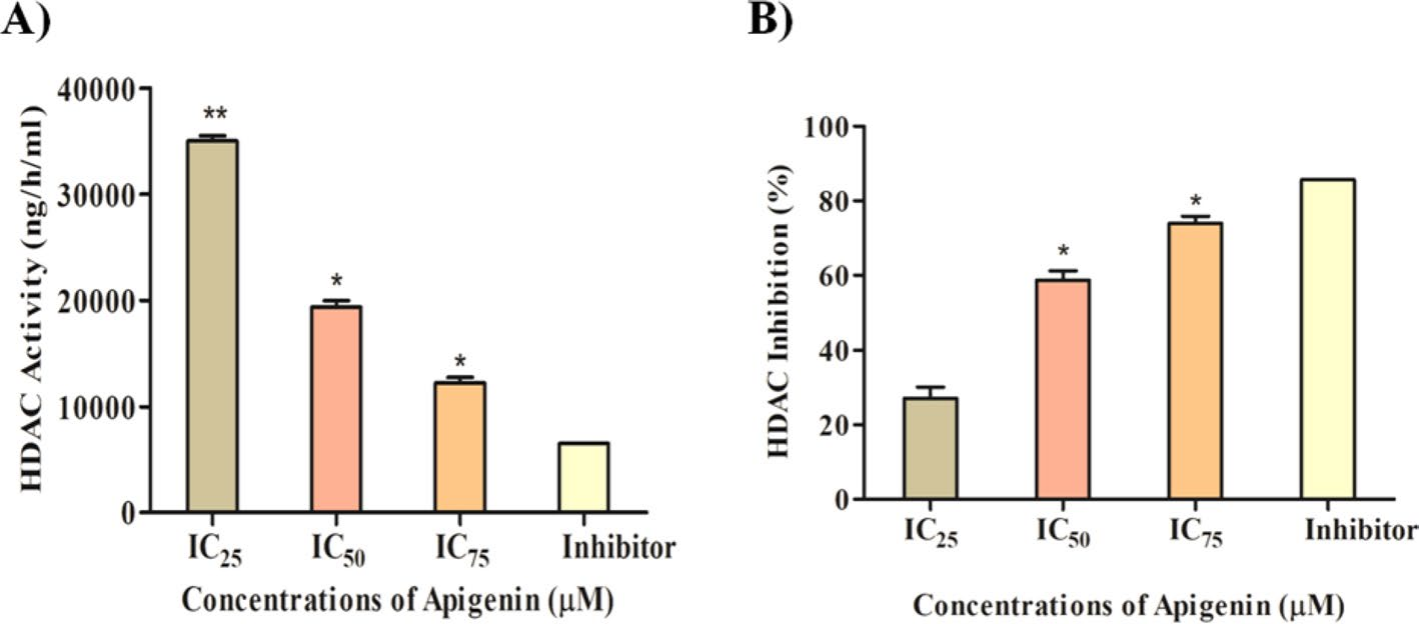
Effect of Apigenin on HDAC activity and inhibition; (**A**) Apigenin decreased HDAC activity in a dose-dependent manner and (**B**) Inhibition of HDAC in a dose-dependent manner.

The enzymatic inhibition of HDACs by Apigenin was determined using an HDAC enzymatic assay kit. In Apigenin-treated MDA-MB-231 cells, the enzymatic activity of HDACs was decreased in a dose-dependent manner (Fig. 11A). At increasing concentrations of Apigenin, more HDACs inhibition was observed in nuclear extract of treated TNBC cells (Fig. 11B).

Apigenin inhibited nearly 60% of HDAC activity at IC_50_ concentration, while more than 75% inhibition was observed at IC_75_ concentration. These results are comparable to the transcriptomic as well as proteomic profiles, where Apigenin downregulated HDAC isoforms in MDA-MB-231 cells. This suggests the significance of Apigenin as an HDAC inhibitor against TNBC cells.

### Apigenin significantly upregulated the HAT activity

**Figure 12:**
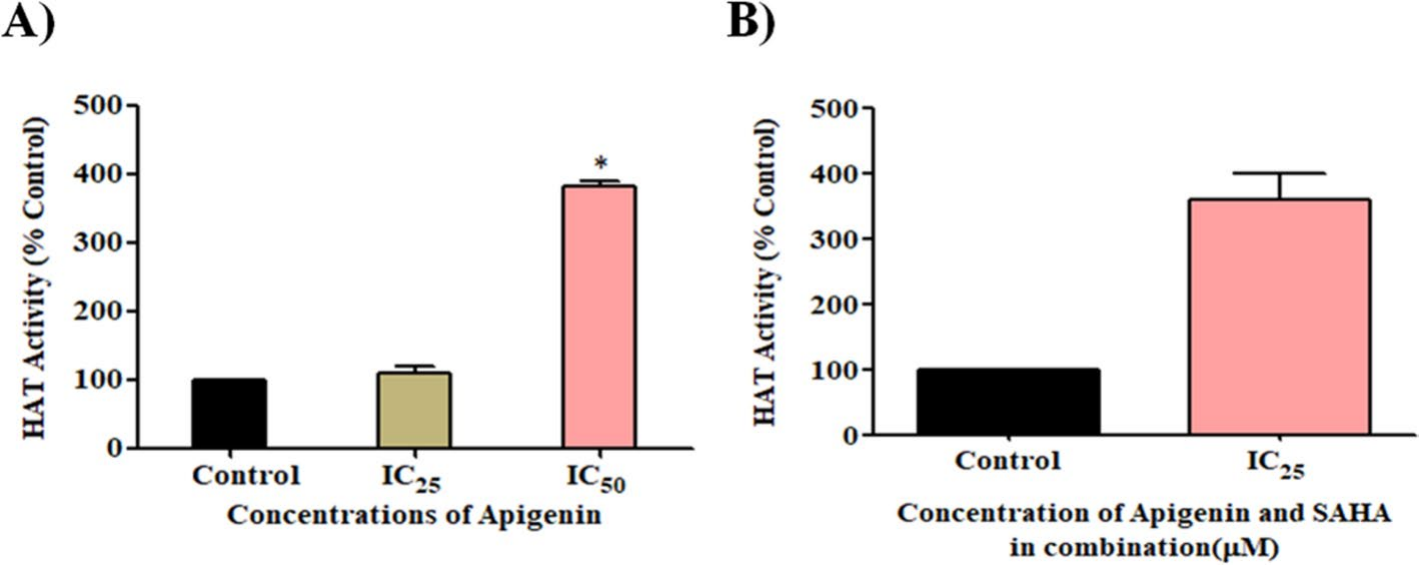
(**A**) Apigenin increased HAT activity in a dose-dependent manner and (**B**) Combination of Apigenin and SAHA increased HAT activity.

In contrast to HDAC activity, Apigenin increased HAT activity in a dose-dependent manner after 48 h of treatment (Fig. 12A). Similarly, HAT activity was increased in the combinatorial treatment of Apigenin and SAHA (Fig. 12B). The results of HAT activity are opposite to HDACs activity, which inhibited by Apigenin in TNBC cells (Fig. 11).

Similar results were obtained in a combinatorial study of Apigenin and SAHA, where at IC_25_ concentration the HAT activity was remarkably increased as compared to control cells (Fig. 12B). Increased HAT activity can be correlated with the inhibition of HDACs activity by Apigenin. Further, increased HAT activity supports the transcriptomic profile of HAT (Fig. 9D). This data suggests that Apigenin can efficiently maintain the equilibrium between HDACs and HAT in MDA-MB-231 cells. The equilibrium between HDACs and HAT is essential for healthy cell growth, but the higher expression of HDACs reduces the HAT expression in cancer cells. Here, we observed HDAC inhibition and increased HAT activity after the treatment of Apigenin and its combination with SAHA. These observations further add the therapeutic significance of Apigenin as an anti-TNBC agent by modulating the expression profiles of epigenetic regulators.

### Apigenin and its combination with SAHA inhibited DNMT activity in MDA-MB-231 cells

**Figure 13:**
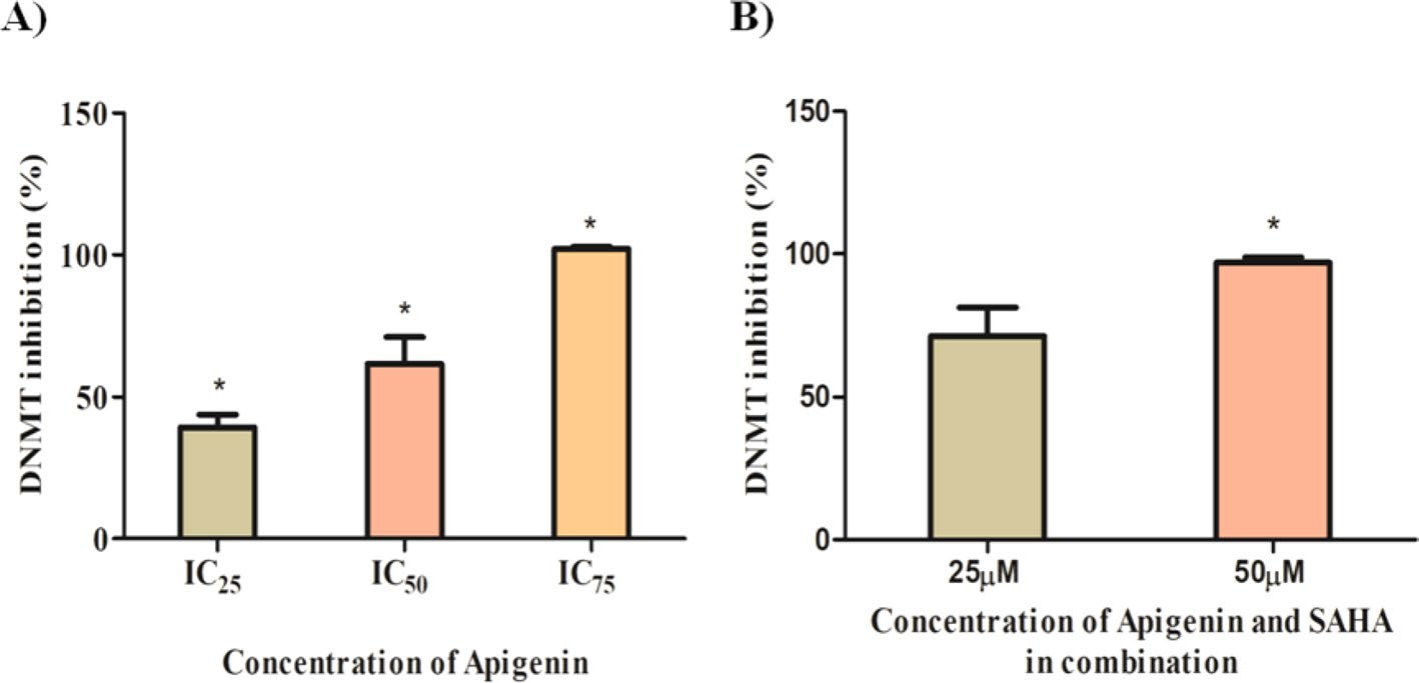
(**A**) Apigenin and (**B**) its combination with SAHA inhibited the activity of DNMT.

The role of Apigenin and its combination with SAHA in DNMT inhibition was investigated by performing DNMT enzymatic kit-based assay. Apigenin inhibited DNMT activity in a dose-dependent manner (Fig. 13A). Nearly 70% of DNMT inhibition was observed at IC_50_ concentration, while total DNMT inhibition was observed at IC_75_ concentration of Apigenin (Fig. 13A). A combinatorial treatment of Apigenin and SAHA significantly inhibited activity of DNMT at IC_25_ and IC_50_ concentrations (Fig. 13B). We anticipated that the drug efficacies of SAHA and Apigenin increased in a synergetic manner.

### Apigenin, SAHA, and their combination modulated the expression of tumour suppressor and onco-miRNAs in MDA-MB-231 cells

**Figure 14:**
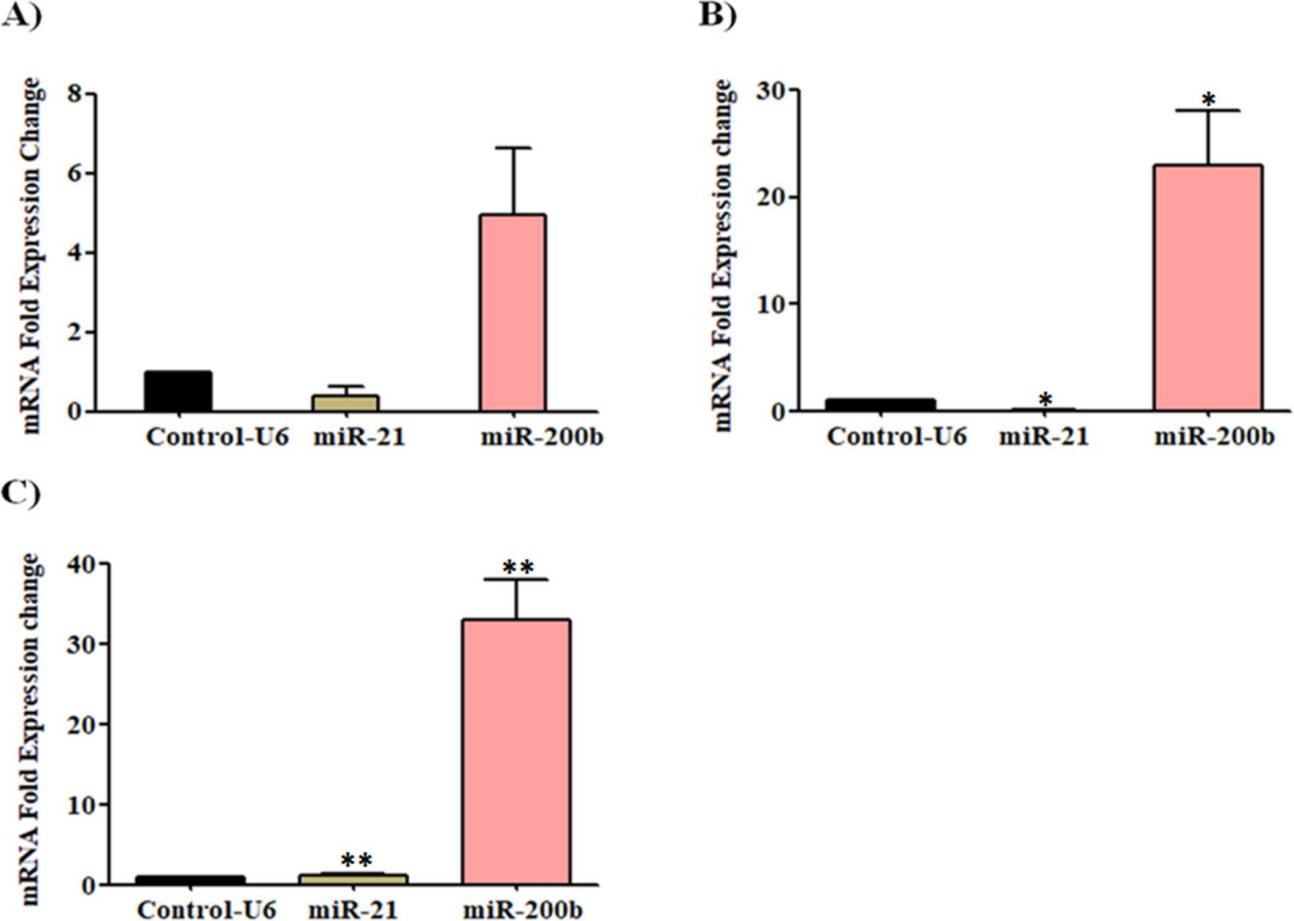
Downregulation of oncomiR-21 and upregulation of tumor-suppressor miR-200 in MDA-MB-231 cells after treatment of (**A**) Apigenin, (**B**) SAHA, and (**C**) combination of Apigenin and SAHA.

The effect of Apigenin, SAHA, and their combination in the regulation of selected and well-known tumour-suppressor and onco-miRNAs has been investigated by performing a qRT-PCR study (Fig. 14).

Apigenin downregulated the expression of oncomiRNA-21 in MDA-MB-231 cells after the treatment of 48 h (Fig. 14A). On the other hand, it increased the expression of tumour suppressor miRNA-200b (Fig. 14A). Similarly, SAHA and its combination with Apigenin downregulated miR-21 and upregulated miR-200b in MDA-MB-231 cells (Fig. 14B,C). The fold expression change in the upregulation of tumour-suppressor miR-200b was higher in SAHA and its combination with Apigenin-treated cells as compared to Apigenin. This observation helps to anticipate the anti-TNBC potential of Apigenin and its combination with SAHA by demonstrating their influence on the expression of tumour-suppressor and oncogenic miRNAs.

### Molecular docking studies of Apigenin and SAHA with HDAC1 and HDAC3

**Figure 15:**
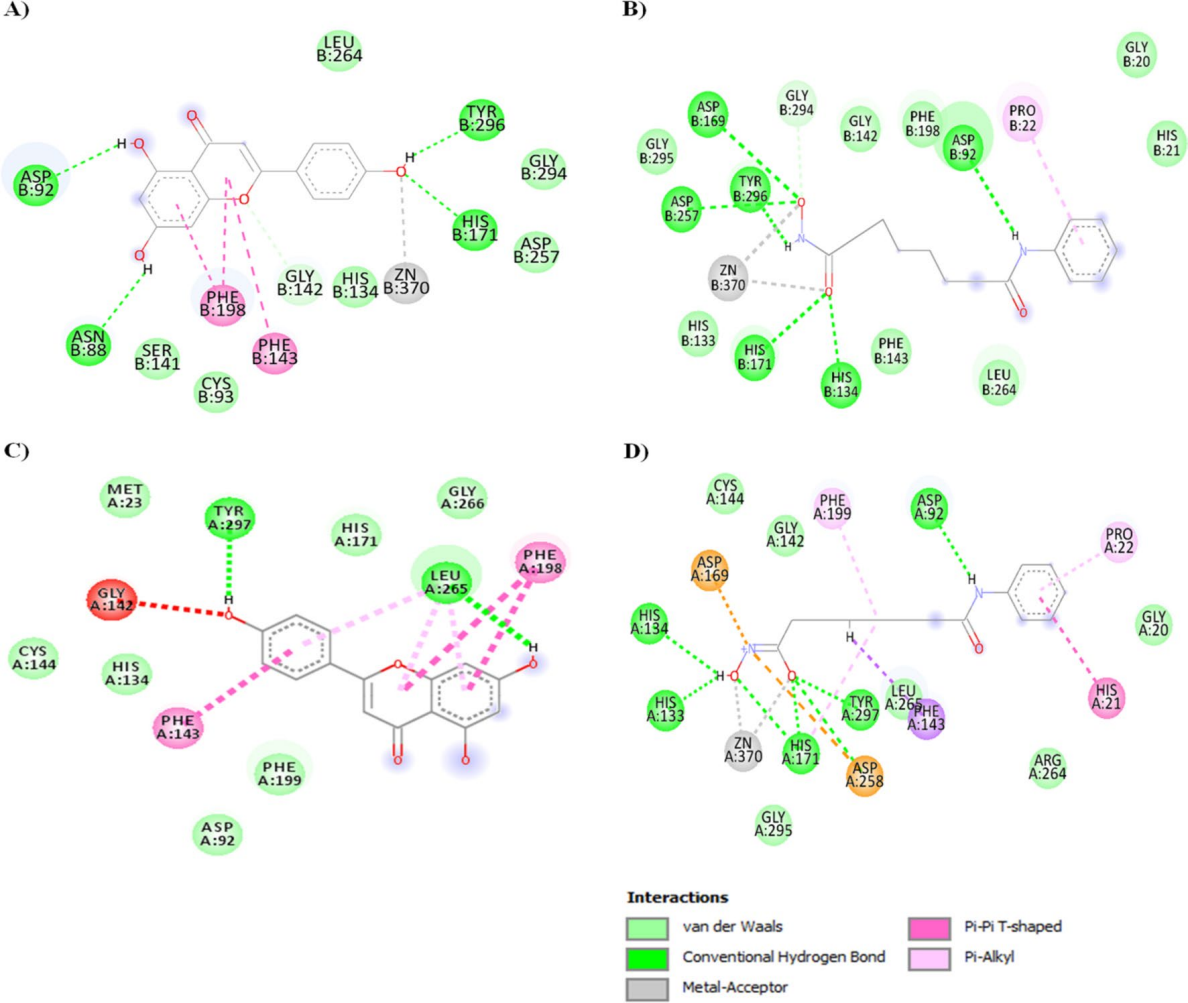
Molecular interactions between favourable docked complexes of (**A**) HDAC1-Apigenin, (**B**) HDAC1-SAHA, (**C**) HDAC3-Apigenin and (**D**) HDAC3-SAHA.

The molecular docking studies of Apigenin and SAHA with HDAC1 and HDAC3 were performed to corroborate the transcriptomic and proteomic profiles and to understand the structural mechanism of catalytic inhibition of HDAC1 and HDAC3. Docking complexes of HDAC1 with Apigenin/SAHA, and HDAC3 with Apigenin/SAHA having the highest gold score and lowest Chemscore were selected for molecular interaction analysis (Fig. 15).

Figure 15 represents the molecular interactions (hydrogen bonds, π–π stacking, metal-coordination, and hydrophobic contacts) between the favourable docked complexes of HDAC1/HDAC3 with Apigenin/SAHA. The geometrical parameters for hydrogen bonding, π–π stacking, Zn^2+^-metal coordination, and hydrophobic interactions are given in Table 1. In the stable docked complex, Apigenin positioned inside the catalytic pocket of HDAC1 in such a way that it would inhibit the catalytic activity of HDAC1 (Fig. 15A). The Asn88, Asp92, His171, and Tyr296 residues from the catalytic pocket of HDAC1 were involved in hydrogen bonding interactions with Apigenin by maintaining 2.99 Å, 1.88 Å, 2.67 Å and 1.68 Å distances, respectively (Table 1). The Phe143 and Phe198 participated in π–π stacking interaction with Apigenin. In this docked complex, Apigenin was strongly coordinated with Zn^2+^, present in the active site pocket of HDAC1 (Fig. 15A). The active site residues including Cys93, His134, Ser141, Cys144, Asp257, Leu264, Gly294, and Gly295 formed hydrophobic contacts with Apigenin (Table 1).

**Table 1:** Summary of molecular interactions between the favourable docked complexes of HDAC1 with Apigenin/SAHA and HDAC3 with Apigenin/SAHA.

HDACs/flavonoid	Atoms involved in H-bonds	Distance (Å)	Atoms involved in H-bonds	Distance (Å)
HDAC1	Apigenin Goldscore = 39.15, Chemscore = − 35.78		SAHA Goldscore = 60.36, Chemscore = − 41.00	
	Api-OH….O-Asn88 Api-OH..O-Tyr296 Api-OH…O-Asp92 Api-O…H-N-His171 Api-O…..Zn^2+^ π–π-stacking …Phe143 π–π-stacking ….Phe198	2.99 1.69 1.88 2.67 2.54	SHH-O…H-Asp92 SHH-O…H-His134 SHH-N–H….O-Asp169 SHH-O…H-His171 SHH-N–H…O-Asp257 SHH-N–H…O-Tyr296 SHH-O…Zn^2+^ π-alkyl….Pro21	2.11 2.43 3.06 2.70 1.82 1.32 1.54
HDAC3	Apigenin Goldscore = 39.27, Chemscore = − 36.39		SAHA Goldscore = 65.13, Chemscore = − 32.89	
	Api-OH…O-Asp258 Api-OH…O-Leu265 Api-O…Zn^2+^ π–π-stacking ….Phe199 π–π-stacking ….Tyr297 π-alkyl…Leu265	2.39 2.00 4.03	SHH-N–H…Asp92 SHH-OH…N-His133 SHH-OH…N-His134 SHH-O…HN-His171 SHH-O…HO-Asp258 SHH-O…HO-Tyr297 SHH-O…Zn^2+^ π–π-stacking ….His21 π-alkyl…Pro22	2.23 1.66 2.97 2.87 3.19 1.94 2.35

Similar interactions were noticed in the molecular docking study of HDAC1 and SAHA (Fig. 15B). Here also, the Asp92, His171, and Tyr296 were involved in hydrogen bonding interactions with SAHA. Additional hydrogen bonding interactions between His134, Asp169, and Asp257 with SAHA were observed in this stable complex (Fig. 15B). The SAHA was coordinated with Zn^2+^ by maintaining a 1.54 Å distance. The Pro22 participated in π–π stacking interaction with SAHA. The Gly20, His21, His133, Gly142, Phe143, Phe198, Leu264, Gly294, and Gly295 were formed hydrophobic contacts with SAHA. The interactions between Apigenin and HDAC1 are comparable to the favourable docking complex of SAHA and HDAC1 (Fig. 15A,B).

Further, in the catalytic pocket of HDAC3, Apigenin was forming hydrogen bonding interactions with Leu265 and Asp258 by maintaining 1.97 Å and 1.94 Å distances, respectively (Fig. 15C and Table 1). Apigenin was forming π–π stacking interaction with Phe199 and Tyr297 residues from the catalytic pocket of HDAC3. The Zn^2+^ was found to be in close contact with Apigenin in the favourable docked complex. The additional stability of Apigenin and HDAC3 complex was expected from hydrophobic interactions between His171, Phe198, Pro200, and Gly266 with Apigenin (Fig. 15C and Table 1). The observed molecular interactions are comparable to the docking complex of reference HDAC inhibitor SAHA and HDAC3 (Fig. 15D). In this docked complex, Apigenin involved in hydrogen bonding interactions with Asp92, His133, His134, His171, and Tyr297 residues from the catalytic pocket of HDAC3. The His21 and Pro22 residues were participated in π–π stacking and π-alkyl interactions with SAHA, respectively. The Gly20, Gly142, Cys144, Arg264, Leu265 and Gly295 were involved in hydrophobic interactions with Apigenin (Fig. 15D). The overall docking results explored the mechanism of catalytic inhibition of HDAC1 and HDAC3 by Apigenin and SAHA in a similar fashion. Further, the docking results are consistent with qRT-PCR, Western blot analysis, and enzymatic inhibition studies of HDACs. These studies suggested the metal (Zn^2+^) dependent inhibition of HDAC1 and HDAC3 by Apigenin.

### Molecular dynamics simulations of HDAC1 and HDAC3 with Apigenin and SAHA

**Figure 16:**
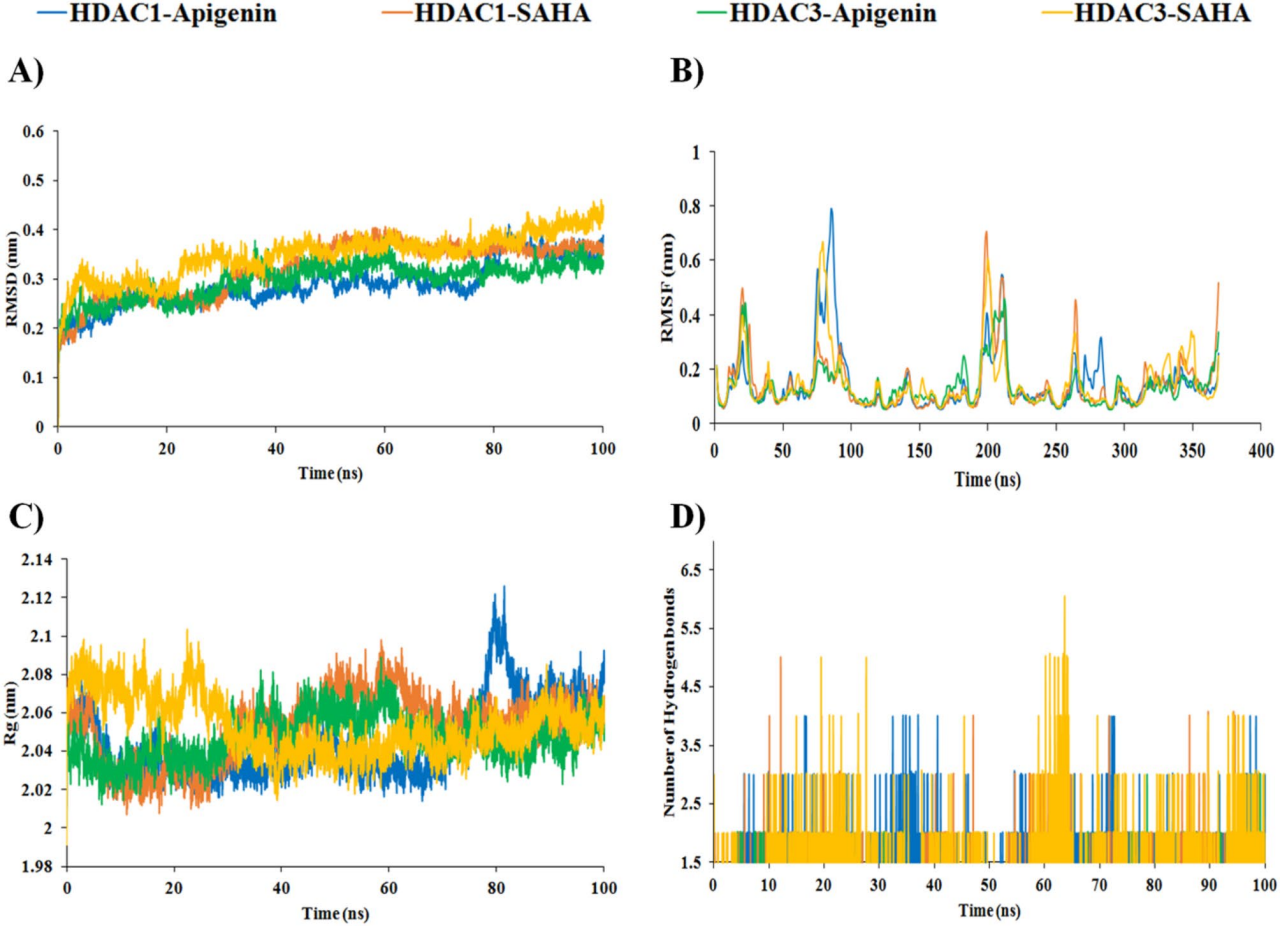
RMSD, RMSF, Radius of gyration and hydrogen bonding interactions from simulated complexes of HDAC1 and HDAC3 with Apigenin and SAHA; (**A**) RMSD profile of HDAC1 and HDAC3 in presence of Apigenin and SAHA, (**B**) RMSF of simulated complexes of HDAC1 and HDAC3 with Apigenin and SAHA, (**C**) Radius of gyration of HDAC1/HDAC3 with bound Apigenin/SAHA and (**D**) Time-dependent hydrogen bonds present during simulation between HDAC1/HDAC3 and Apigenin/SAHA.

Molecular dynamics simulations of 100 ns were performed on the stable docked complexes of HDAC1 and HDAC3 with Apigenin and SAHA to investigate the effect of explicit water solvent on the stability of docked complexes (Fig. 16). The MD simulation results were analysed to assess the binding affinity of the Apigenin and SAHA towards the HDAC1 and HDAC3. The conformational stability of docked complexes of HDAC1/HDAC3 with Apigenin/SAHA was investigated by calculating the root mean square deviation (RMSD) and root mean square fluctuations (RMSF) of the protein backbone atoms and side chain residues, respectively (Fig. 16A,B). The averaged RMSD values for simulated complexes of HDAC1 with Apigenin and SAHA are 0.28 nm and 0.32 nm, respectively (Fig. 16A). Similarly, the average RMSD of HDAC3 with Apigenin and SAHA ranged 0.29–0.34 nm during 100 ns of simulations (Fig. 16A). RMSD values of all simulated complexes are below 0.35 nm, which indicated the overall stability of Apigenin and SAHA in the catalytic pocket of HDAC1 and HDAC3 (Fig. 16A).

The RMSF values of all simulated complexes of HDAC1/3 with Apigenin/SAHA are below 0.3 nm, which signifies the good stability of simulated complexes (Fig. 16B). The catalytic residues participated in hydrogen bonding, π-stacking and hydrophobic interactions with Apigenin and SAHA showed the least residual (< 0.14 nm) fluctuations during 100 ns of simulations. Large fluctuations in RMSF (0.2–0.75 nm) were observed in the loop regions of HDAC1/HDAC3 during simulations. Further, the compactness of HDAC1 and HDAC3 in the presence of Apigenin and SAHA was predicted by calculating the radius of gyration (Rg) (Fig. 16C). The Rg values (2.0–2.1 nm) of simulated complexes of HDAC1/HDAC3 with Apigenin/SAHA represent the compactness of HDAC1 and HDAC3 due to the stable behaviour of secondary structures during simulations. Figure 16D depicts the intermolecular hydrogen bonding interactions between the simulated complexes of HDAC1/HDAC3 with Apigenin/SAHA during 100 ns of simulations.

**Figure 17:**
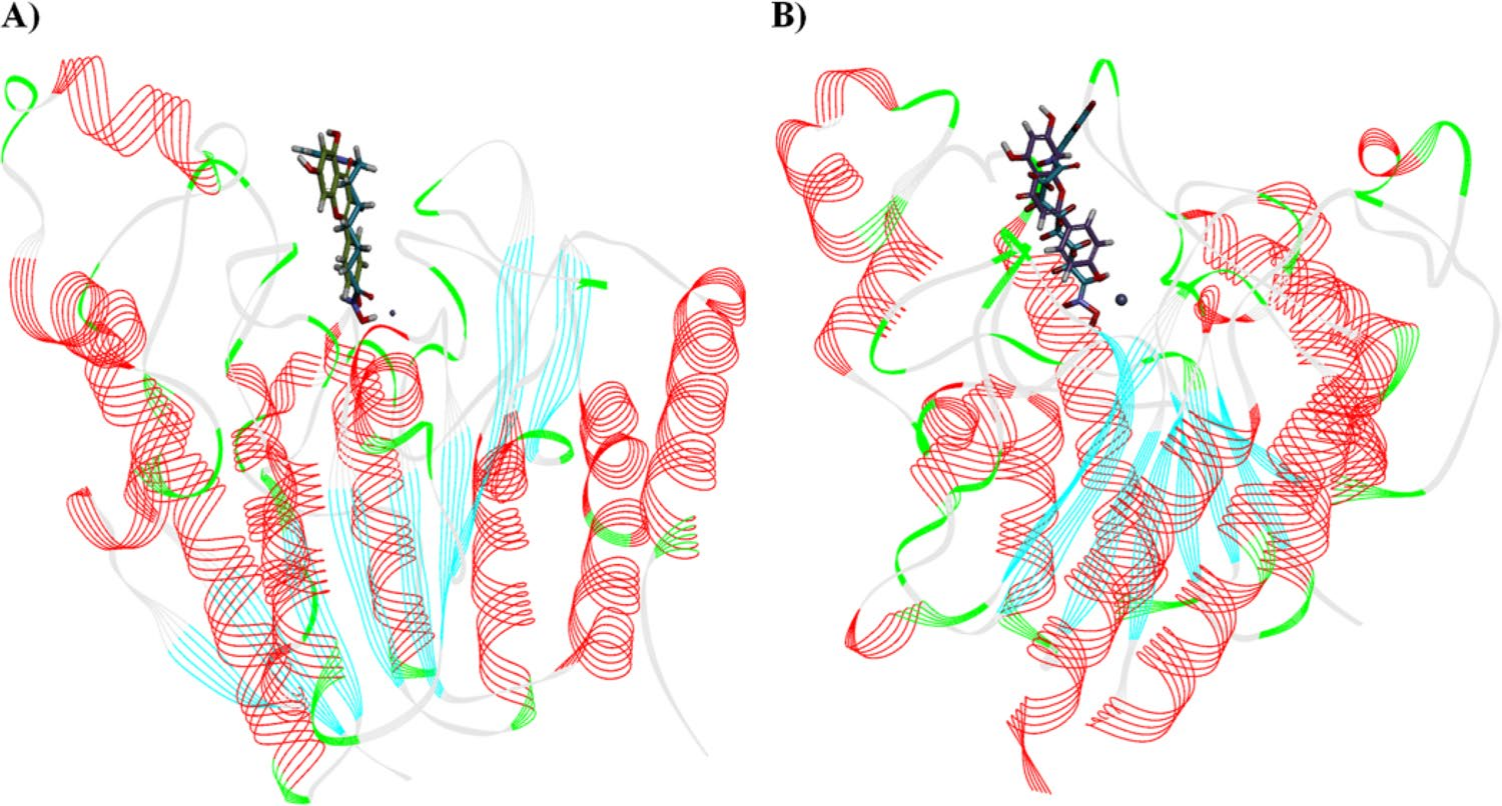
(**A**) Apigenin and SAHA bound in the deep catalytic cavity of HDAC1, and (**B**) Apigenin and SAHA bound in the deep catalytic cavity of HDAC3.

Further, the binding mode of Apigenin and SAHA within the catalytic pocket of HDAC1 and HDAC3 was studied by superimposing the representative structures of simulated complexes (Fig. 17). The Apigenin and SAHA were superimposed in the catalytic pocket of HDAC1 and HDAC3 by maintaining a similar binding pattern and strong coordination with the catalytic Zn^2+^ (Fig. 17).

**Figure 18:**
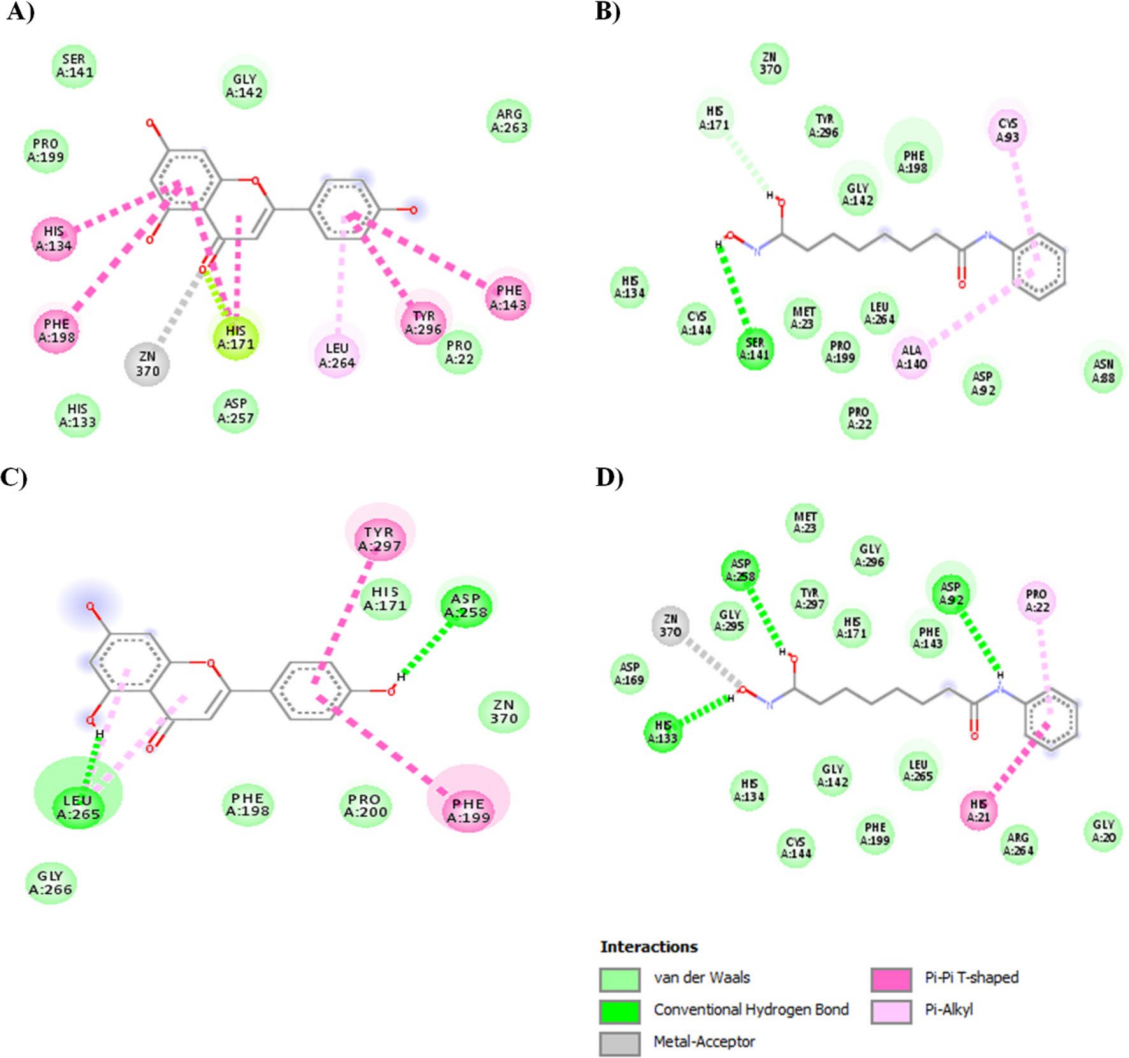
Simulated complex of HDAC1 with Apigenin, (**B**) SIMULATED complex of HDAC1 with SAHA, (**C**) simulated complex of HDAC3 with Apigenin, and (**D**) simulated complex of HDAC1 with SAHA.

The simulated complexes of HDAC1 with Apigenin/SAHA and HDAC3 with Apigenin/SAHA were analysed for intermolecular hydrogen bonds, π–π stacking, Zn^2+^-metal coordination, and hydrophobic interactions (Fig. 18, Table 2). In simulated complexes, Apigenin was involved in π-stacking interactions with His134, Phe143, Phe198, and Tyr296 active site residues from the catalytic pocket of HDAC1 (Fig. 18A). Apigenin also formed strong coordination with catalytic Zn^2+^ by maintaining 2.97 Å distance (Fig. 18A). The His171 participated in π-lone pair interaction with Apigenin. The additional stabilization of the simulated complex of HDAC1 with Apigenin was expected from hydrophobic interactions between Pro22, His133, Ser141, Gly142, Pro199, Asp257, Arg263, and Leu264 residues from the active site pocket of HDAC1.

**Table 2:** Molecular interactions between simulated complexes of HDAC1 and HDAC3 with Apigenin and SAHA.

Molecule name	Atoms involved in H-bonds	Distance (Å)	Hydrophobic and Van der walls contacts	Fig. Ref
Api-HDAC1	Api-O3….. Zn^2+^	2.97	Pro22, His133, Ser141, Gly142, Pro199, Asp257, Arg263, and Leu264	Figure 18A
	π–π-stacking ….His134	-		
	π–π-stacking ….Phe143	-		
	π–π-stacking ….His171	-		
	π–π-stacking ….Phe198	-		
	π–π-stacking ….Tyr296	-		
SHH-HDAC1	SHH-OH…O-Ser141	2.58	Pro22, Met23, Asn88, Asp92, Cys93, His134, Ala140, Gly142, Cys144, Phe198, Pro199, Leu264, and Tyr296	Figure 18B
	SHH-OH…His171	2.65		
	π-alkyl….Cys93	-		
	π-alkyl….Ala140	-		
Api-HDAC3	Api-OH…O-Asp258	2.39	His171, Phe198, Pro200 and Gly266	Figure 18C
	Api-OH….O-Leu265	2.00		
	SHH-O…Zn^2+^	4.03		
	π–π-stacking ….Phe199	-		
	π–π-stacking ….Tyr297	-		
	π-alkyl…Leu265	-		
SHH-HDAC3	SHH-N–H…Asp92	2.45	Gly20, His21, Pro22, Met23, His134, Gly142, Phe143, Cys144, Asp169, His171, Phe199, Arg264, Leu265, Gly295, and Gly296	Figure 18D
	SHH-OH…N-His133	2.13		
	SHH-O…HO-Asp258	2.60		
	SHH-O…Zn^2+^	2.92		
	π–π-stacking ….His21	–		
	π-alkyl…Pro22	–		

Similarly, SAHA maintained a stable complex with HDAC1 during 100 ns of MD simulation study (Fig. 18B). SAHA was involved in hydrogen bonding interaction with Ser141 from the active site pocket of HDAC1 by maintaining a 2.57 Å distance. The Cys93 and Ala140 were involved in π-alkyl interactions with SAHA. The hydrophobic contacts from Pro22, Met23, Asn88, Asp92, Cys93, His134, Ala140, Gly142, Cys144, Phe198, Pro199, Leu264, and Tyr296 with SAHA were expected to provide additional stability to the simulated complex during 100 ns (Table 2).

The simulated complex of HDAC3 with Apigenin was stabilized by hydrogen bonding, π–π stacking, Zn^2+^ coordination, and hydrophobic interactions (Fig. 18C). The Asp258 and Leu265 catalytic residues were involved in hydrogen bonding interactions with Apigenin by retaining 2.39 Å and 2.0 Å distances, respectively (Fig. 18C and Table 2). Apigenin participated in π–π stacking interactions with Phe199 and Tyr297 catalytic residues from the active site pocket of HDAC3. Apigenin also maintained close contact with catalytic Zn^2+^ during the simulation. In addition, Apigenin formed hydrophobic contacts with His171, Phe198, Pro200, and Gly266 residues from the active site pocket (Table 2). Similarly, SAHA maintained a stable complex with HDAC3 during 100 ns of simulation by maintaining hydrogen bonding interactions with Asp92, His133, and Asp258 catalytic residues (Fig. 18D). The π–π stacking and π-alkyl interactions between His21 and Pro22 residues with SAHA were observed during simulation, respectively. SAHA was coordinated with catalytic Zn^2+^ by maintaining a 2.91 Å distance. The additional stability of SAHA and HDAC3 complex was expected from hydrophobic contacts between Gly20, His21, Pro22, Met23, His134, Gly142, Phe143, Cys144, Asp169, His171, Phe199, Arg264, Leu265, Gly295, and Gly296 residues (Fig. 18D, Table 2). The hydrogen bonding, π-interactions, Zn^2+^-metal coordination, and hydrophobic contacts from simulated complexes of Apigenin with HDAC1/HDAC3 are comparable to that of SAHA with HDAC1/HDAC3. The simulation data supported the qRT-PCR and western results of HDAC inhibition by Apigenin, and thus, anticipated the similar mechanism of catalytic inhibition of HDAC1/HDAC3 by Apigenin and SAHA. Therefore, Apigenin can be used as an anti-TNBC agent by modulating the expression of epigenetic regulators.

### Binding free energy calculations using the MM-PBSA method

The binding mechanism and binding proficiency of Apigenin and SAHA toward the HDAC1 and HDAC3 were investigated by calculating the MM-PBSA (Table 3). Total binding energy (ΔG_binding_) of Apigenin-HDAC1, SAHA-HDAC1, Apigein-HDAC3 and SAHA-HDAC3 are − 74.208 ± 17.217, − 122.936 ± 10.647, − 92.168 ± 38.275 and − 106.486 ± 22.615, respectively.

**Table 3:** The binding free energy (kJ/mol) between simulated complexes of Apigenin and SAHA with HDAC1 and HDAC3 calculated by using the MM-PBSA method.

Complex	ΔE_vdw_	ΔE_elec_	ΔG_polar_	ΔG_non-polar_	ΔG_binding_
Api-HDAC1	− 157.55 ± 15.38	− 10.11 ± 8.80	107.14 ± 24.15	− 13.67 ± 1.40	− 74.20 ± 17.21
SAHA-HDAC1	− 175.63 ± 12.76	− 20.62 ± 7.52	90.64 ± 14.64	− 17.32 ± 1.16	− 122.93 ± 10.64
Api-HDAC3	− 138.56 ± 18.10	− 3.80 ± 5.46	64.41 ± 42.81	− 14.20 ± 1.78	− 92.16 ± 38.27
SAHA-HDAC3	− 138.92 ± 21.34	− 6.26 ± 10.50	53.02 ± 12.77	− 14.32 ± 1.52	− 106.48 ± 22.61

**∆**Δ_Evdw_, ∆E_ele_, ∆Δ_Gpolar_, ∆G_non-polar,_ and ∆G_binding_ represented van der Waals energy, electrostatic energy, polar solvation energy, nonpolar solvation energy, and binding energy, respectively.

**Figure 19:**
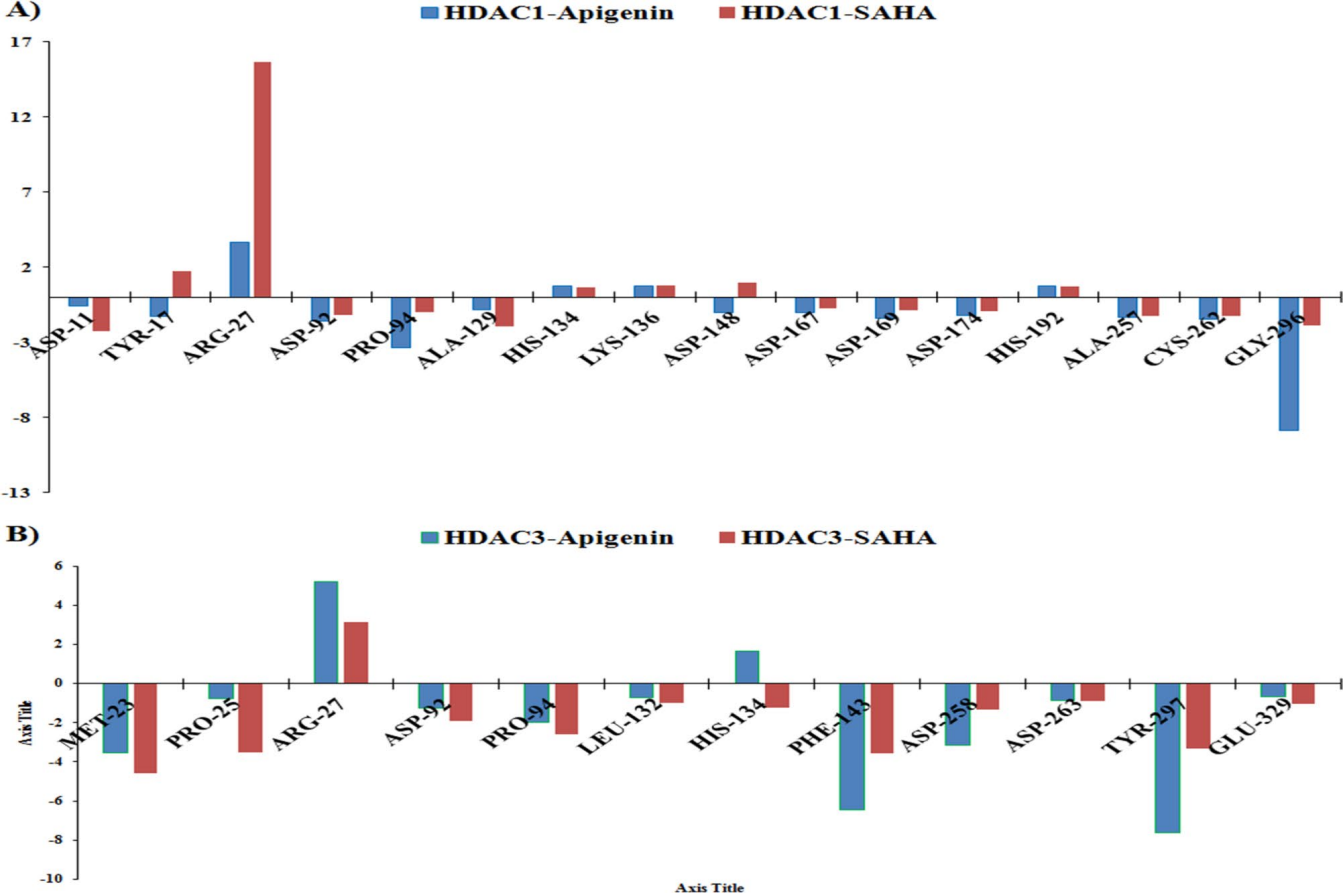
Energetic contribution of individual residues from simulated protein–ligand complexes of (**A**) Apigenin and HDAC1 (Blue color) and SAHA and HDAC1 (Red color). (**B**) Apigenin and HDAC3 (Blue color) and SAHA and HDAC1 (Red color).

This suggested that Apigenin and SAHA have a strong binding affinity toward HDAC1 and HDAC3. The VDW energy (ΔE_vdw_) has a major contribution to the total binding energy and is more favourable for the complex formation between Apigenin with HDAC1 and HDAC3. Similarly, ∆G_non-polar_ energy was good for the complex formation between Apigenin and SAHA with HDAC1 and HDAC3. This signifies that ΔE_vdw_ and ∆G_non-polar_ energies are favourable for the binding of Apigenin/SAHA to HDAC1/HDAC3. The total binding energies showed stability of SAHA-HDAC1/HDAC3 complexes as compared to Apigenin-HDAC1/HDAC3. In addition, the residual contribution of each active site residue of HDAC1 and HDAC3 in complex formation with Apigenin and SAHA was estimated by calculating the residual binding free energy decomposition using the MM-PBSA (Fig. 19).

The Asp11, Tyr17, Asp92, Pro94, Ala129, Asp148, Asp167, Asp169, Asp174, Ala257, Cys262, and Gly296 residues from HDAC1 were involved in binding with Apigenin and SAHA (Fig. 19A). The Met23, Pro25, Asp92, Pro94, Leu132, His134, Phe143, Asp258, Asp263, Tyr297, Glu329 residues of HDAC3 were involved in binding with Apigenin and SAHA (Fig. 19B). The Asp92, His134, Phe143, Asp258, and Tyr297 were involved in hydrogen bonding and π–π stacking interactions with Apigenin and SAHA. These residual interactions supported the binding energy and favourable binding of Apigenin and SAHA with HDAC1 and HDAC3. The molecular docking interactions preserved in simulation studies supported the inhibition of HDAC1 and HDAC3 by Apigenin observed at transcriptomic and proteomic levels. Moreover, these interactions are in good agreement with the reference drug SAHA. Therefore, Apigenin may act as a potent inhibitor of HDAC1 and HDAC3 in a fashion like SAHA and can be used as an anti-cancer agent for the treatment of TNBC patients.

## Discussion

The extensive heterogeneity and absence of hormonal receptors in triple-negative breast cancer leads to more aggressive and invasive tumour growth^4^. Also, the lack of specific target-oriented drug therapy and high levels of cancer recurrence are reducing the overall survival rate in TNBC patients^5^. At the chromatin level, the epigenetic regulators (HDACs, HAT, and DNMTs) control the regulation of tumour suppressors and oncogenes that preclude effective cancer treatment^6,7^. In addition, the severe and unbearable side effects produced by anticancer agents worsen the targeted therapies and reduce the overall success rate of cancer treatment^53^. To overcome these issues and to get a better treatment option against proliferative and very aggressive cancer subtypes, researchers are using plant flavonoids alone and in combination with other anticancer drugs, and become one of the most significant cognitive aspects^56–58,110–114^.

The anticancer role of dietary flavonoid 'Apigenin' has been well documented using different molecular targets except for its interventions in epigenetic modulations in the invasive TNBCs^66–70^. The role of Apigenin in modulating epigenetic regulators and inducing apoptosis-mediated cell death was understood using MDA-MB-231 cells. Apigenin has displayed potent anti-TNBC activity at the individual level and in combination with a reference anticancer drug 'vorinostat' by inducing adverse morphological changes. A more abundant and intact spindle-shaped morphology and basal level of attachment were noticed in control cells. In contrast, Apigenin-treated cells had a less intact and rounded morphology, and cells were detached from the surface. These results may help to anticipate the initiation of programmed cell death pathways in TNBC cells. Similarly, morphological changes have been noticed in the combinatorial treatment of 5-fluorouracil and gelam honey in human adenocarcinoma colon cancer^113^. This combination works in a synergetic manner by reducing individual IC50­ concentrations and increasing the drug efficacy of 5-fluorouracil toward MDA-MB-231 cells. The dietary flavonoids are well known for their promising anticancer activities and for demonstrating synergetic effects on cancer cell lines alone and in combination with FDA-approved drugs^63,110–112^. The metastatic potential of cancer is associated with the migration ability of cancer cells. The inhibition of wound closing and cell migration ability of Apigenin and its combination with SAHA is like an earlier study performed on prostate cancer using the combination of metformin and quercetin^112^. Therefore, Apigenin and its combination with SAHA may help to reduce the metastatic potential of TNBC cells.

Further, the generation of reactive oxygen species and a decrease in mitochondrial membrane potential in cancer cells represents the symbolic link between the anticancer potential of flavonoids and apoptotic-mediated cell death in various human cancers^12,114–116^. The generation of ROS and reduction in mitochondrial membrane potential after the treatment of Apigenin may lead to the release of cytochrome-C from mitochondria to cytosol that activates the caspase-3/procaspase-9 and induces the apoptotic-mediated cell death in MDA-MB-231 cells^12^. The high DNA damage and reduced nutrient supply initiate the stress phenotype that generates ROS in cancer cells. This alters the redox homeostasis and exerts oxidative stress that induces cell damage and apoptotic-mediated cell death in TNBCs. Further, DNA damage, nuclear fragmentation, and chromatic condensations were noticed in DAPI staining after the treatment of Apigenin, SAHA, and a combination of Apigenin with cisplatin^117^. These observations explored the anticancer potential of Apigenin. A similar mechanism of other flavonoids in various cancer subtypes has represented a shred of concrete evidence for the anticancer activity of Apigenin.

The flavonoids have been reported for their most significant potential to arrest cells at subG0/G1 phases of the cell cycle and induce apoptotic-mediated cell deaths in various human cancers^27,118,119^. The higher population of MDA-MB-231 cells arrested at subG0/G1 phases in the combinatorial treatment of Apigenin and SAHA by synergetic mechanism. The apoptotic-mediated cell death population was higher in Apigenin-treated cells compared to SAHA and a combinatorial treatment of Apigenin and SAHA. However, the cell necrosis was more in SAHA and a combinatorial treatment of Apigenin and SAHA. These observations can highlight the significance of Apigenin as an apoptotic inducer in cancer cells. The FACS analysis corroborates the apoptotic-mediated cell death mechanism anticipated using DCFDA assay, DAPI staining and JC-1-based mitochondrial membrane potential assays.

The epigenetic regulators HDACs and DNMTs play a crucial role in various cellular cascades. Therefore, they are considered prime targets for drug discovery against malaria, Leishmania, and various neurological disorders^39^. In this direction, the role of flavonoids in modulating epigenetic regulators such as HDACs, HAT, and DNMTs has been studied enormously in different cancer subtypes at transcriptomic and proteomic levels. Only a few reports have explored the role of Apigenin in the modulation of HDAC expression in prostate cancer^120^. Combinatorial treatment of Apigenin and SAHA has downregulated all HDAC isomers, whereas Apigenin and SAHA upregulated HDAC5/9/10 and HDAC2 at individual levels, respectively. The downregulation of DNMT and upregulation of HAT were observed in all treatments. On the same line, the EGCG has inhibited the expression of DNMT1 and prevented the methylation of genes during the S-phase of the cell cycle^48^. The maintained equilibrium between the HDAC and HAT expression levels is necessary for the natural growth and the usual functioning of normal cells. Here also, a synergetic effect of Apigenin and SAHA was observed that is in close agreement with an earlier study^48^.

In addition to epigenetic regulators, the anti- and pro-apoptotic markers are associated with a more aggressive phenotype, invasive tumour growth, and elevated metastasis potential in cancer cells. The flavonoids induced apoptosis by upregulating the pro-apoptotic markers and downregulating the anti-apoptotic proteins^78,112,115,121,122^. Here also, Apigenin, SAHA, and their combinations downregulated the anti-apoptotic marker (Bcl2 and Nrf2) and upregulated the pro-apoptotic (p53, Cas3/cas8, Bax, and Bid) markers in MDA-MB-231 cells. This data again helps to anticipate the role of Apigenin in the induction of apoptotic-mediated cell deaths in TNBC cells. The downregulation of epigenetic regulators (HDAC1/HDAC3), anti-apoptotic markers (Bcl2 and NRF2), and upregulation of pro-apoptotic markers (Bax, Bak, Bid, Caspase-9, and PARP) at proteomic level governed the accuracy of transcriptomic results. Similar results have been reported in earlier transcriptomic and proteomic studies of flavonoids against various cancers^112^. The equilibrium phenomenon of upregulation of pro-apoptotic markers and downregulation of anti-apoptotic markers suggested induction of apoptotic-mediated cell deaths in TNBC cells by Apigenin and its combination with SAHA. This supports the conclusive remark that Apigenin acts as a potent anti-TNBC agent by inducing apoptotic-mediated cell death and modulating epigenetic and apoptotic regulators in MDA-MB-231 cells. The increase in cleaved products of caspase-3 and caspase-9 in Apigenin-treated cells confirms the caspase-mediated activation of the apoptosis pathway^112^. The Poly-ADP-Ribose polymerase (PARP) is a widely used pro-apoptotic marker to identify the induction of the apoptosis process in drug-treated cancer cells^117^. Here also, the increase in the cleaved product of PARP anticipated the role of Apigenin in the induction of apoptosis in TNBCs.

The enzymatic inhibition of HDAC/DNMT and activation of HAT supported transcriptomic and proteomic profiling of epigenetic regulators. The increase in HAT activity leads to activation and restoration of transcription of tumour suppressor genes by increasing acetylation of H3K9 and H3K27 of histone H3^123^. The DNMT-activated aberrant methylation pattern potentiates the events of tumour occurrence in many human cancers^124^. The downregulation of DNMT at the transcriptomic level and its enzymatic inhibition supported the anti-TNBC potential of Apigenin. Cancer proliferation and high tumour invasion have also associated with elevated expressions of oncogenic miRNAs and suppression of tumour suppressor miRNAs^80–83^. After the treatment of Apigenin, SAHA, and their combination on MDA-MB-231 cells, the onco-miRNA-21 and tumour-suppressor miRNA-200b were downregulated and upregulated, respectively. The miRNA-21 is associated with EMT and cell migration in cancer cells. The downregulation of miRNA-21 leads to a reduction of migration and metastasis in the MDA-MB-231 cells. The responsive correlation between epigenetic regulators and miRNA expression has been well investigated in many flavonoid-treated cancer cells^60^. The molecular docking of ligands with the target protein has emerged as a very effective tool in modern drug development practices^125,126^. Molecular docking studies have been performed to understand the most appropriate conformations and binding modes of hit compounds with target proteins, small molecular interactions with catalytic site residues, and their binding affinities^127–129^. The MD simulations have been extensively used to understand the mechanism of catalytic inhibition of various molecular targets by different flavonoids^79^. In line with earlier reports, the performed molecular docking explored the role of molecular interactions in catalytic inhibition of HDAC1 and HDAC3 by Apigenin and SAHA^79,130^. A similar mode of action was observed for Apigenin and SAHA against the HDAC1 and HDAC3 by maintaining docking interactions in simulation studies. The calculated RMSD, RMSF, and radius of gyration supported the stability of simulated complexes between Apigenin/SAHA with HDAC1/HDAC3. The MMPBS also explored the energetic role of active site residues in binding to Apigenin and SAHA and supported the docking results. Therefore, based on these observations, we propose a similar mode of action for both SAHA and Apigenin against TNBC cells by modulating epigenetic and apoptotic regulators and inducing apoptotic-mediated cell death in MDA-MB-231 cells. Therefore, Apigenin and its combination with SAHA may be a suitable strategy to treat aggressive tumour growth in TNBC patients.

## Conclusion

Apigenin displayed a potent anti-TNBC activity at IC50 = 49.9 μM by inducing adverse morphological changes in MDA-MB-231 cells. Apigenin has generated high levels of reactive oxygen species, reduced mitochondrial membrane potential, and arrested the cell cycle at subG0/G1 phases. This thereby induced apoptotic-mediated cell death in TNBC cells. Apigenin significantly modulated the expression profile of epigenetic regulators by downregulating HDAC and DNMT and upregulating HAT activity. Apigenin has induced apoptotic-mediated cell deaths in MDA-MB-231 cells by upregulating the pro-apoptotic markers (p53, Bax, Bak, Bid, Caspase3/8/9, and PARP) and downregulated anti-apoptotic (Bcl2 and Nrf2) proteins in TNBC cells. Apigenin increased the expression of tumour-suppressor miR-200b and decreased the expression of oncomiR-21. Apigenin inhibited HDAC/DNMT activity and increased HAT activity in Apigenin-treated TNBC cells. A combination of Apigenin and SAHA inhibited the proliferation of MDA-MB-231 cells in a synergetic manner. These results are in line with the standard HDAC inhibitor SAHA. From the experimental and in-silico observations, we anticipated that the mode of action of Apigenin and SAHA is the same against TNBC cells. Therefore, this study may help to design an effective apigenin-mediated strategy to treat the more aggressive and metastatic conditions of TNBCs.

### Supplementary Information

Supplementary Information.

## Abbreviations

TNBC: Triple-negative breast cancer
DNMT: DNA methyl transferase
HAT: Histone acyl transferase
IC_50_: Inhibitory concentration
NRF: Nuclear factor erythroid 2-related factor 2
miR: MicroRNA
PR: Progesterone receptor
ER: Estrogen receptor
HER2: Human epidermal growth factor receptor 2
VEGF: Vascular endothelial growth factor
PD1: Programmed cell death protein 1
CTLA-4: Cytotoxic T-lymphocyte-associated antigen HDACi-HDAC inhibitor
PARP: Poly (ADP-ribose) polymerases
PTEN: Phosphatase and TEN sin homolog deleted on chromosome 10
SAHA: Suberoylanilide hydroxamic acid
EMT: Epithelial to mesenchymal transition
TNF-α: Tumour necrosis factor alpha
BCRP: Breast cancer resistance protein
CM-H_2_: DCFDA-5-(and-6)-chloromethyl-2′,7′-dichlorofluorescein diacetate acetyl ester
FITC: Fluorescein isothiocyanate
MTT-3: (4,5-Dimethylthiazol-2-yl)-2,5-diphenyltetrazoliumbromide
HRP: Horseradish peroxidase
DMSO: Dimethyl sulphoxide
ROS: Reactive oxygen species
DAPI: 4',6-Diamidino-2-phenylindole
PBS: Phosphate buffer saline
FACS: Fluorescence-activated cell sorting
PVDF: Polyvinylidene fluoride
TBST: Tris Buffer saline containing tween
GOLD: Genetic optimization for ligand docking
GROMACS: Groningen machine for chemical simulations
LINCS: Library of integrated network based cellular signatures
PME: Particle mesh Ewald
RMSF: Root mean square fluctuations
Rg: Radius of gyration
Zn^2+^: Zinc
PBSA: Poisson–Boltzmann solvent accessible surface area
Ps: Picosecond
MD: Molecular dynamics
