# Supplementary material for: Apigenin and its combination with Vorinostat induces apoptotic-mediated cell death in TNBC by modulating the epigenetic and apoptotic regulators and related miRNAs
Source: Sci Rep. 2024 Apr 25;14:9540. doi: 10.1038/s41598-024-60395-x (PMC11045774; doi:10.1038/s41598-024-60395-x)
Supplement: Supplementary file 1 — Supplementary Information. [file 41598_2024_60395_MOESM1_ESM.docx]

**Supplementary Information**

**Apigenin and its combination with Vorinostat induces apoptotic-mediated cell death in TNBC by modulating the epigenetic and apoptotic regulators and related miRNAs**

Snehal Nimal,^a^ Navanath Kumbhar,^a,b^ Saruchi,^a^ Shriya Rathore,^a^ Nitin Naik,^c^ Sneha Paymal,^c^ Rajesh Gacche.^1^*

a Department of Biotechnology, Savitribai Phule Pune University Pune, Maharashtra (MS),

411007, India.

b Medical Information Management, Department of Biochemistry, Shivaji University,

Kolhapur, Maharashtra (MS) 416004, India.

c Department of Microbiology, Shivaji University, Kolhapur, Maharashtra (MS) 416004,

India.

***Corresponding Address:**

Professor Rajesh N Gacche,

Department of Biotechnology,

Savitribai Phule Pune University (SPPU), Pune, India, 411007.

Email.: [rngacche@unipune.ac.in](mailto:rngacche@unipune.ac.in), [rngacche47@gmail.com](mailto:rngacche47@gmail.com)

Tel.: +91-20-25694952

Fax.: +91-20-25692248

**Table 1 SI: List of primers used for mRNA and miRNA expression analysis using RT-qPCR**

| **GENE NAME** | **FORWARD PRIMER** | **REVERSE PRIMER** |
| --- | --- | --- |
| BAX | GACATGTTTTCTGACGGCAAC | AAGTCCAATGTCCAGCCC |
| BCL2 | GTGGATGACTGAGTACCTGAAC | GCCAGGAGAAATCAAACAGAGG |
| BID | ATTAACCAGAACCTACGCACC | TGACCACATCGAGCTTTAGC |
| Caspase 3 | ACTGGACTGTGGCATTGAG | GAGCCATCCTTTGAATTTCGC |
| Caspase 8 | CTGGGAGAAGGAAAGTTGGAC | GAGTCCGAGATTGTCATTACCC |
| HDAC1 | GGTGCTGGACATATGAGACAG | AAGGACTGATGTGGAGCTTG |
| HDAC2 | CTTCCAGCTTCTCTTGTATCCTC | ATTGCCTGTCCGTACTTTCC |
| HDAC3 | CCCAGACTTCACACTTCATCC | GGTTCAGCATCTTCAGGTTTTC |
| HDAC4 | GGAATCTGAACCACTGCATTTC | GAACTCTGGTCAAGGGAACTG |
| HDAC5 | CTTTAAGATGCAGAGTGGAGGG | CAGAGATGGTGAGGACGAAAG |
| HDAC6 | ATCCAAGTCCATCGCAGATAC | TCTTTTCTCGTGTGGTCATCC |
| HDAC7 | GCAGATCATTCAACAGCCATG | TTGGTAGAAGGTTTGCTGGG |
| HDAC8 | GCATTCAGTTTCACCTCCAAAG | AACATCAGACACGTCACCTG |
| HDAC9 | TTCTGCCTCACCATTCTCTTC | CTGGCGACCTCTTAACTTCTC |
| HDAC10 | GTCCTTTACTTCTCCTGGCAC | GTAGTCAGCGTTTCCCATCC |
| HDAC11 | GTCTACAACCGCCACATCTAC | GTTCCTCTCCACCTTATCCAG |
| GAPDH | ATGACTCTACCCACGGCAAG | CTGGAAGATGGTGATGGGTT |
| DNMT1 | AAGCCAGCTATGCGACTTGGAAAC | ACAACCGTTGGCTTTCTGAGTGAG |
| HMT | AAAACCCAAATGCCGTGGTG | TCAGCAAATGAGCCCAGAAG |

| miR21-5p RT specific | GTCGTATCCAGTGCAGGGTCCGAGGTATTCGCACTGGATACGACTCAACAT |
| --- | --- |
| miR21 Forward Primer | TGTCCGCCTAGCTTATCAGAC |
| miR200b RT specific | GTCGTATCCAGTGCAGGGTCCGAGGTATTCGCACTGGATACGACTCCAAT |
| miR200b Forward Primer | AACAGTGCATCTTACTGGGCA |
| RNU6B RT specific | GTCGTATCCAGTGCAGGGTCCGAGGTATTCGCACTGGATA CGACAAAAATATGG |
| RNU6B Forward Primer | TGCGGCTGCGCAAGGATGA |
| Universal Reverse primer | GTGTCGTGGAGTCGGCAATTC |

**Raw images of Western Blots**

**HDAC1**


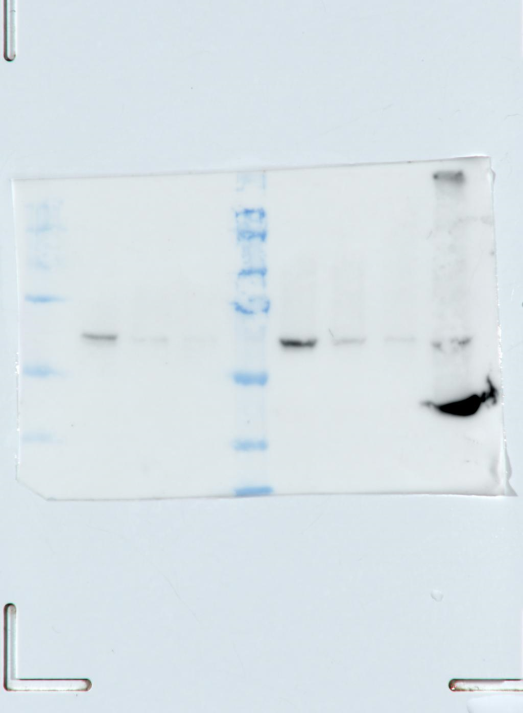

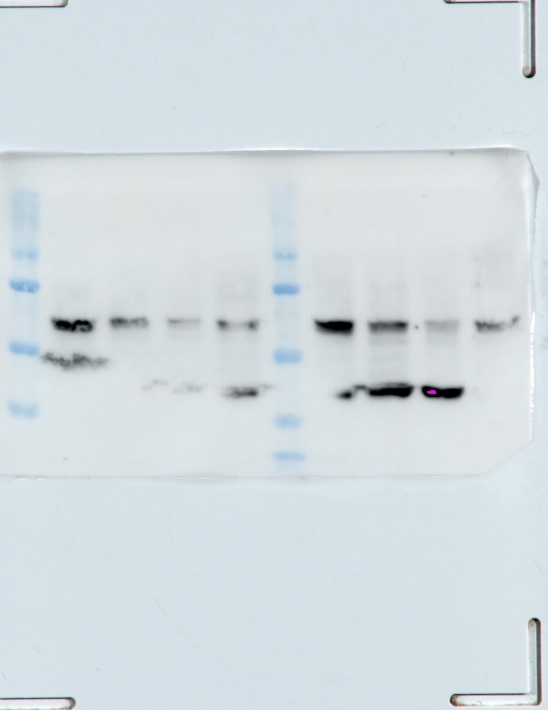


62 KDa

**Actin**


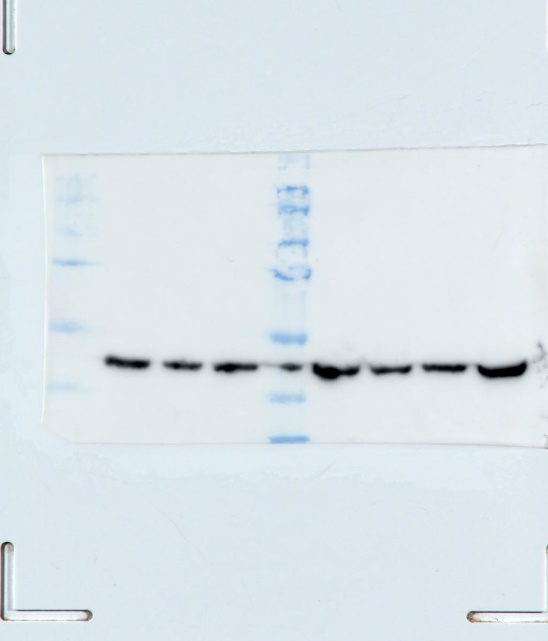

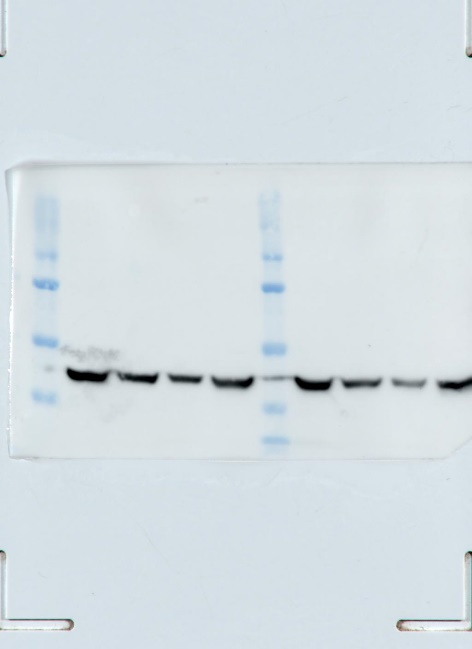


43 KDa

**HDAC3**


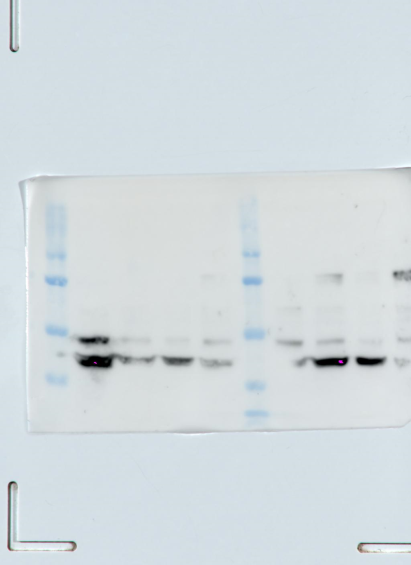

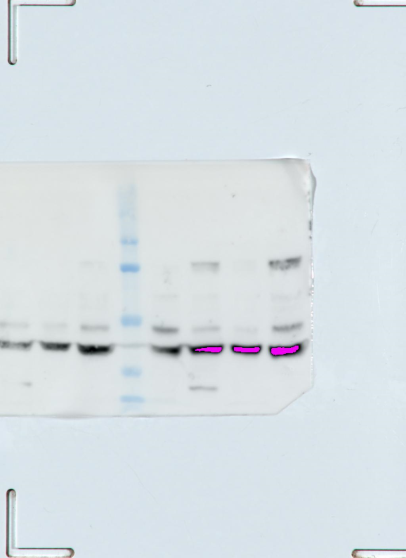

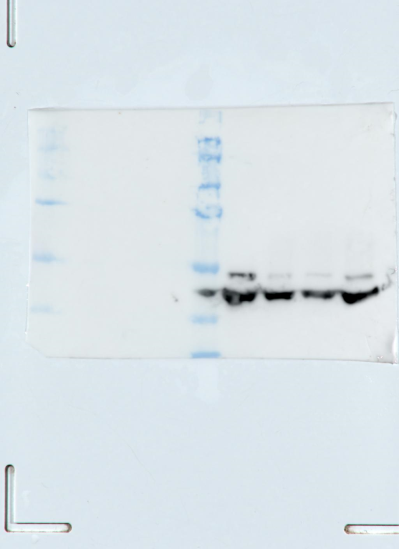


49 KDa

**Actin**


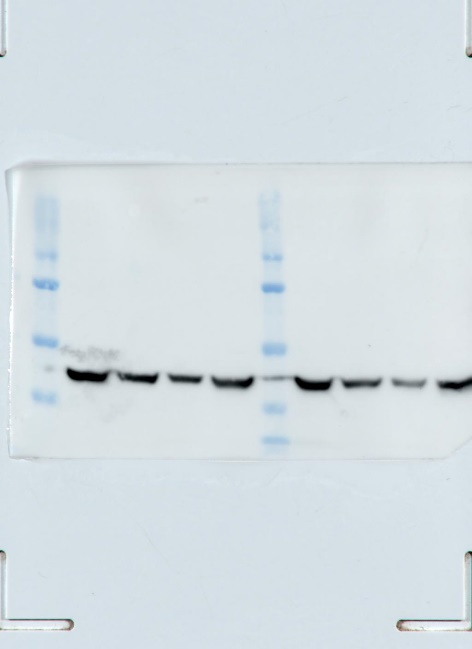

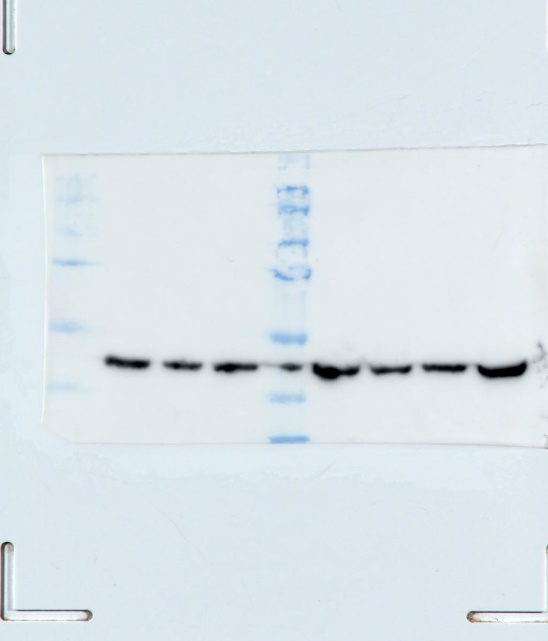


43 KDa

**
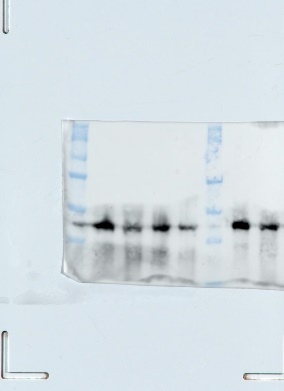
BCL (18%)**


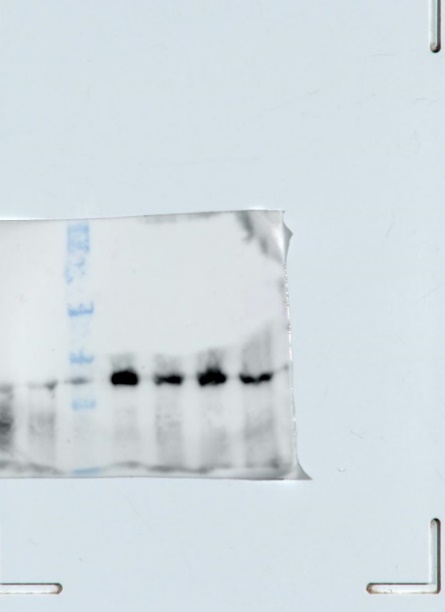


26 KDa

**Actin (BCL, BAX, BID and BAK)**


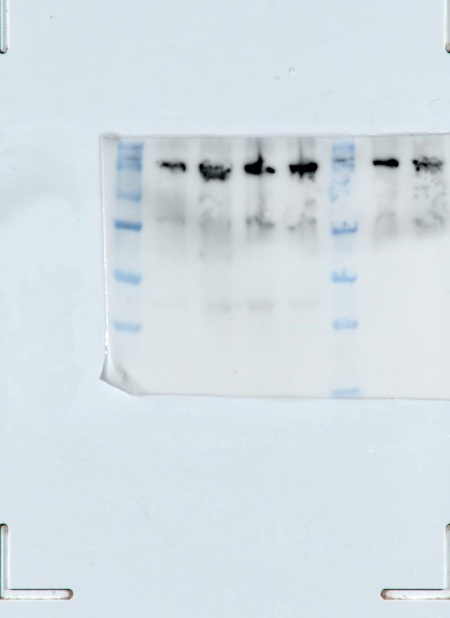

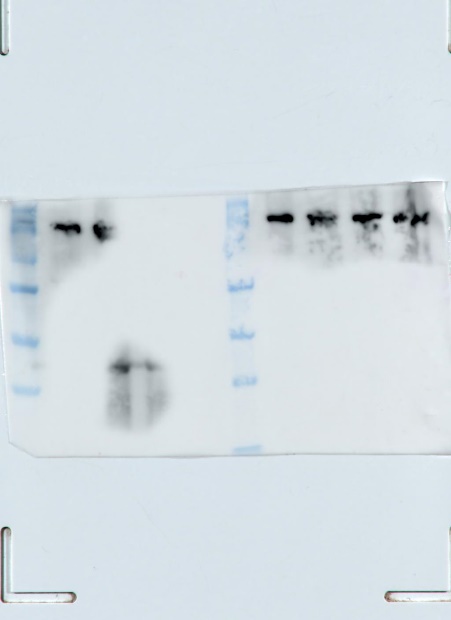


43 KDa

**BAX**


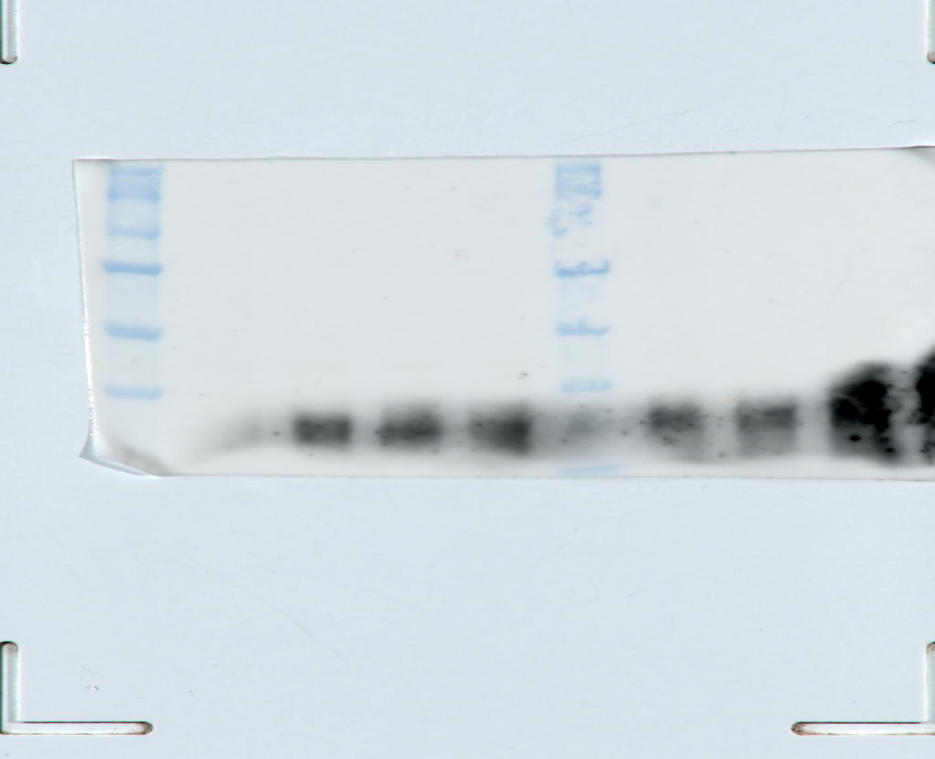


23 KDa

23KDa

**BID**


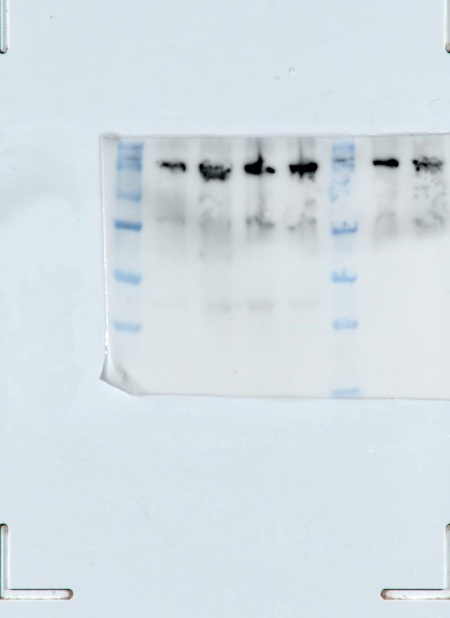

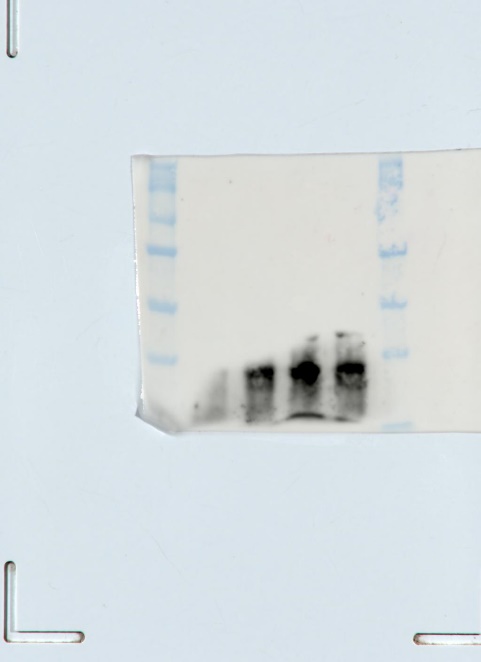

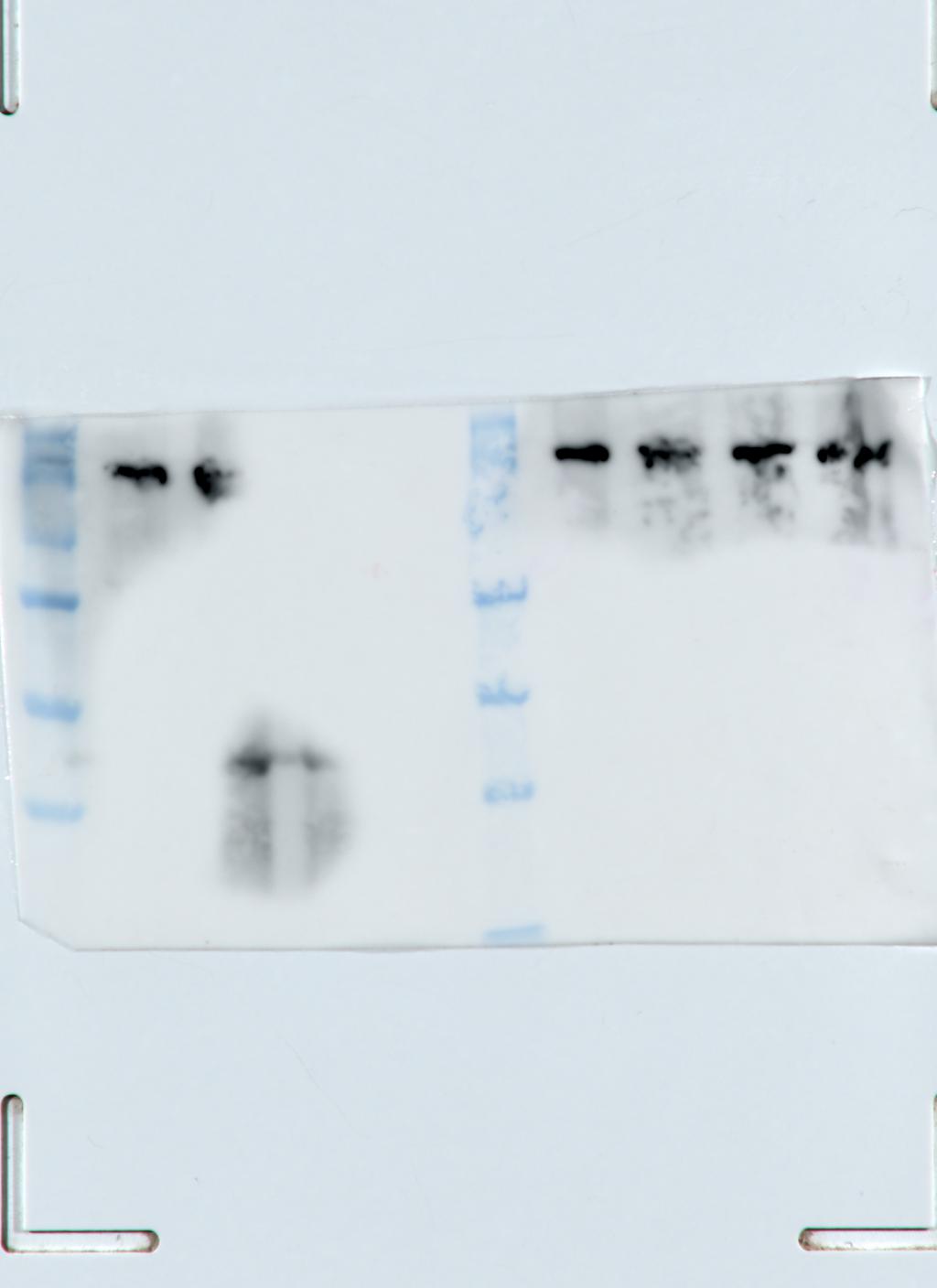

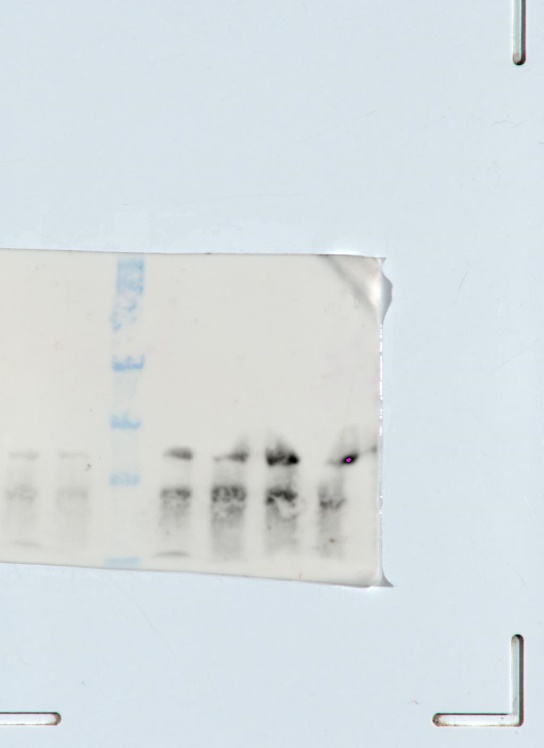


22 KDa

22 KDa

Actin (43 KDa)

Actin (43 KDa)

**
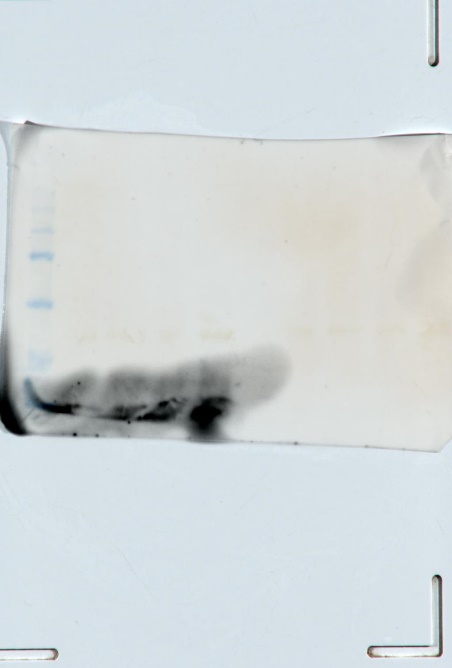
BAK**


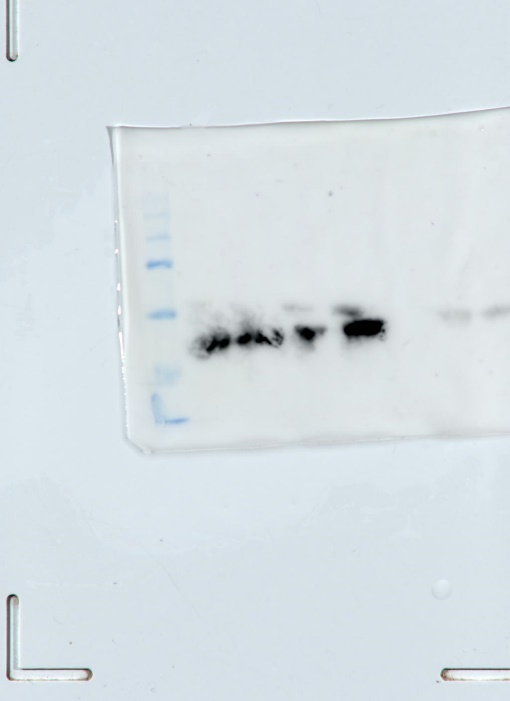

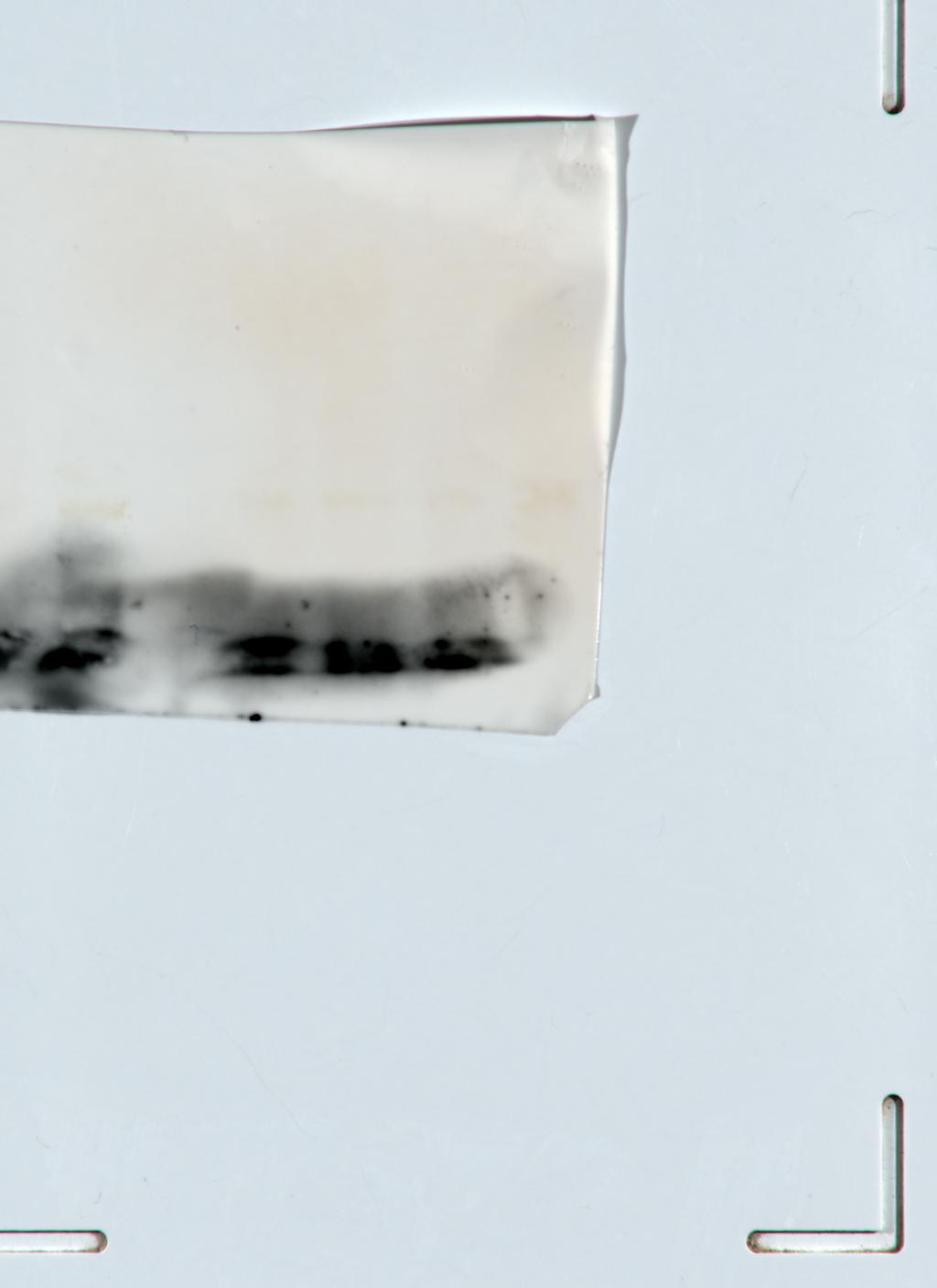

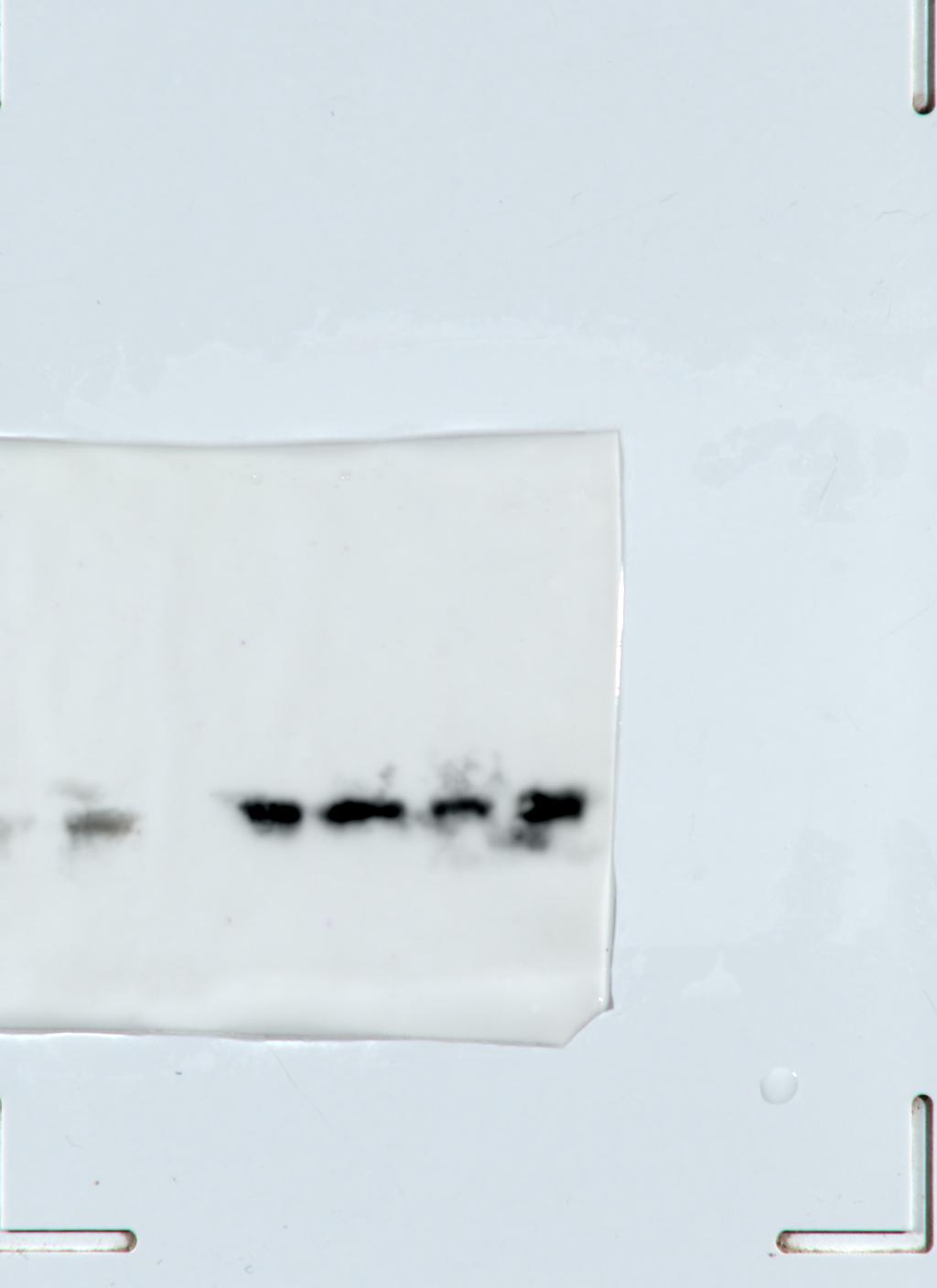


24 KDa

Actin 43 KDa

Actin 43 KDa

24 KDa

**Caspase 9**


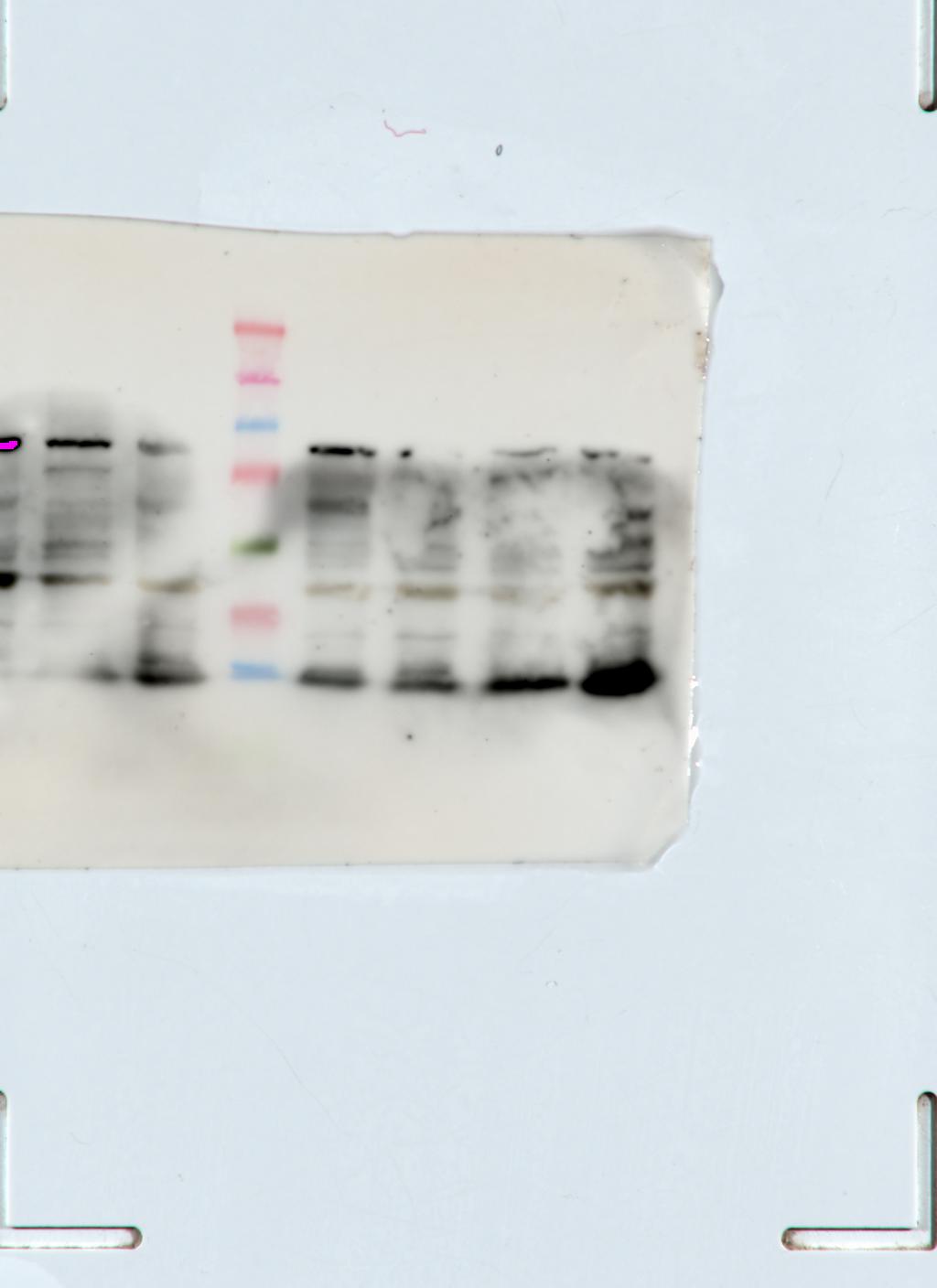

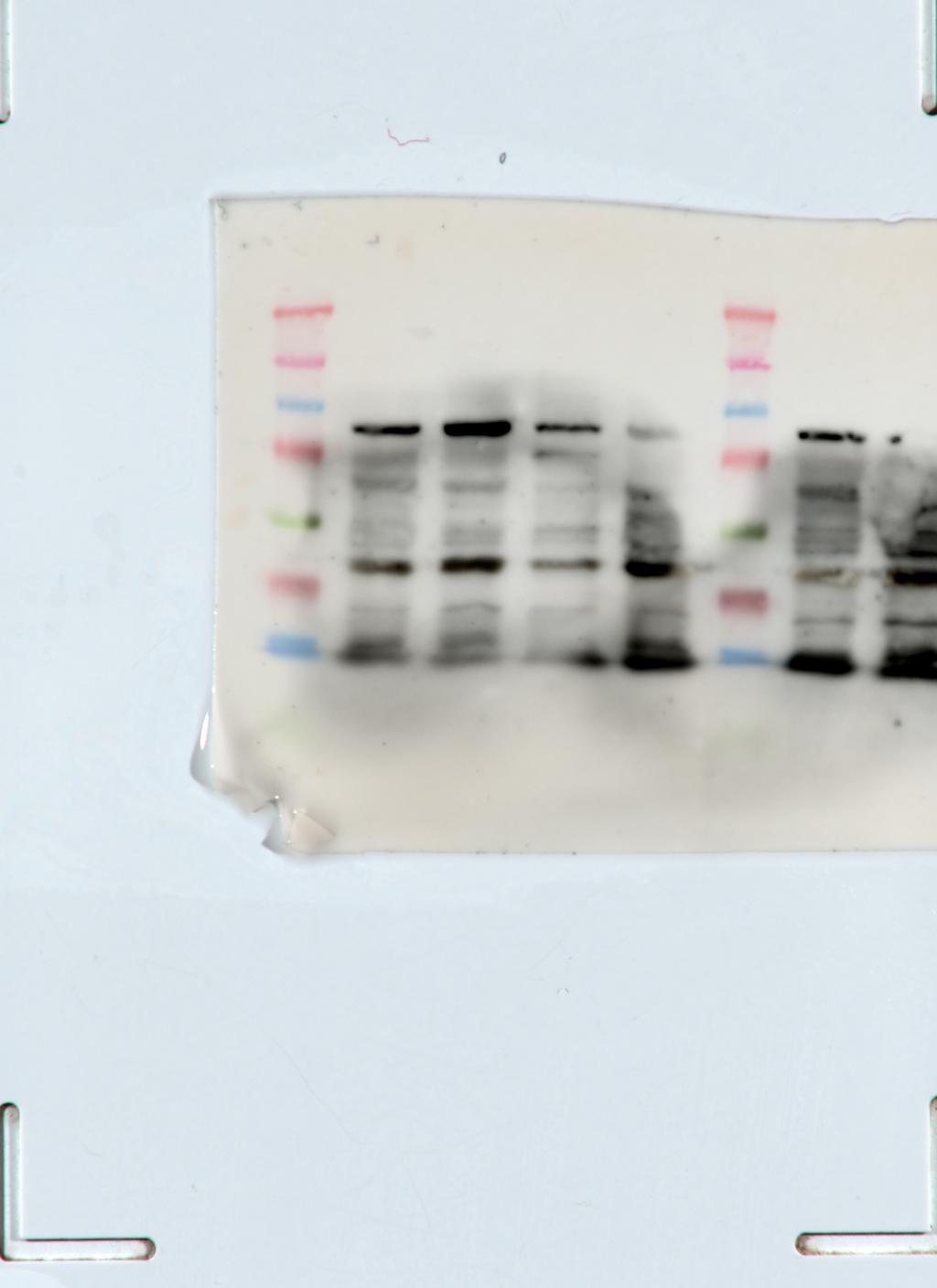


35 KDa

46 KDa

46 KDa

35 KDa

**Actin**


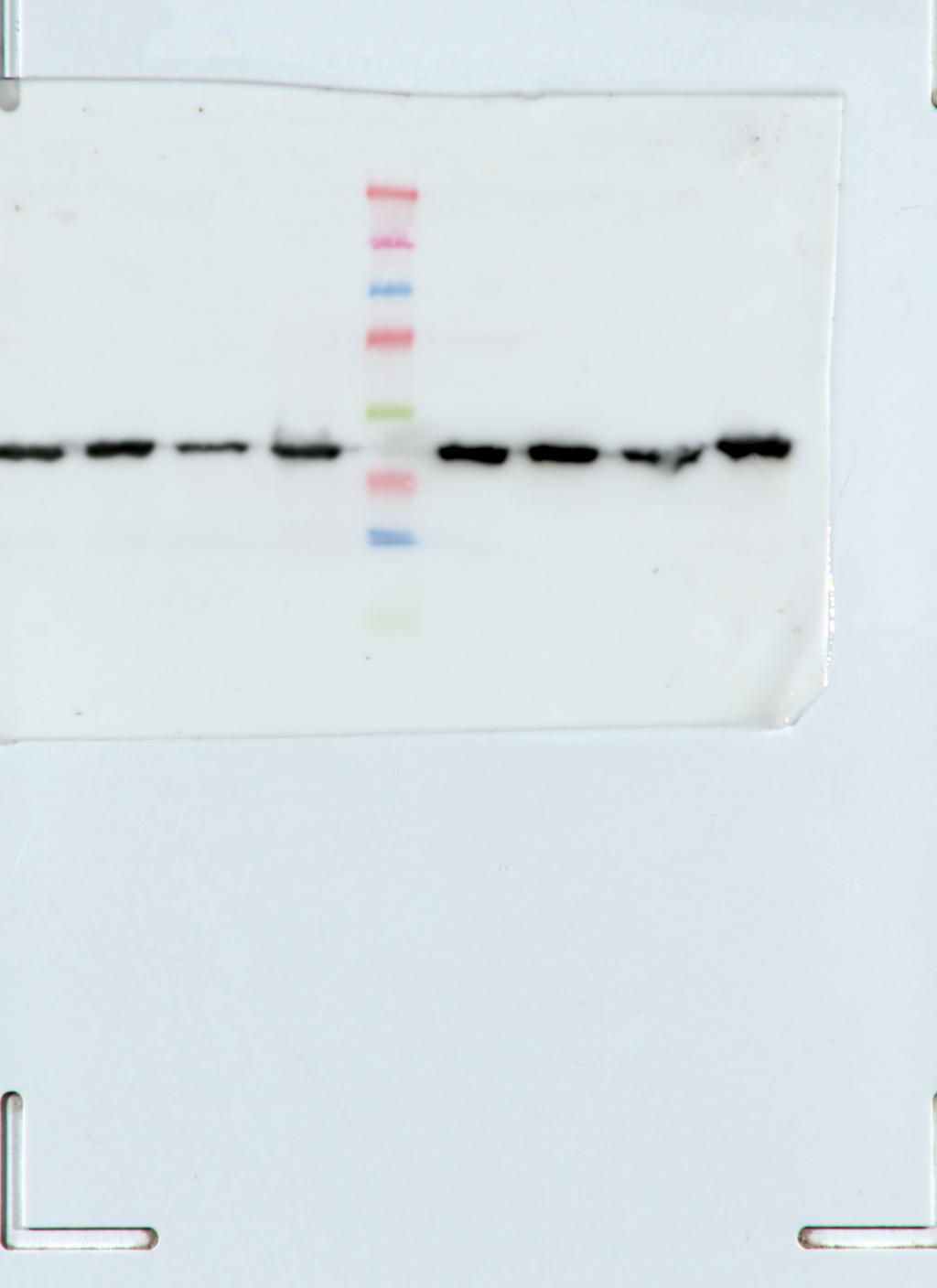


43 KDa

**Caspase 9**


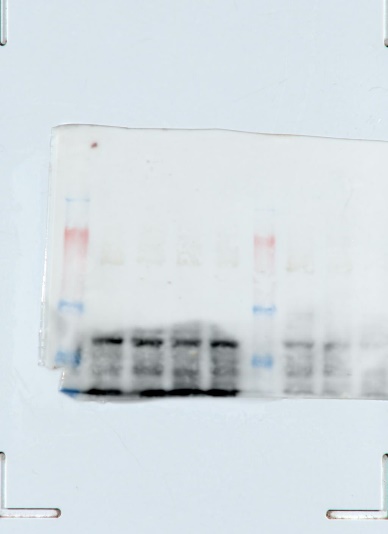

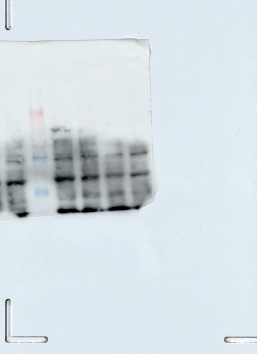


46 KDa

35 KDa

46 KDa

35 KDa

**Actin**


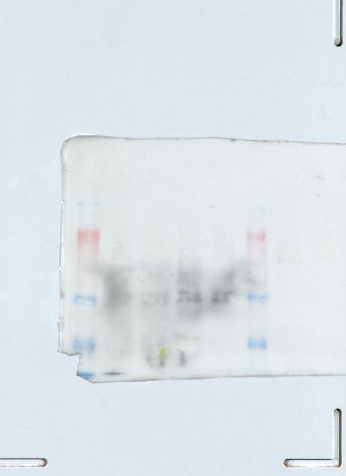

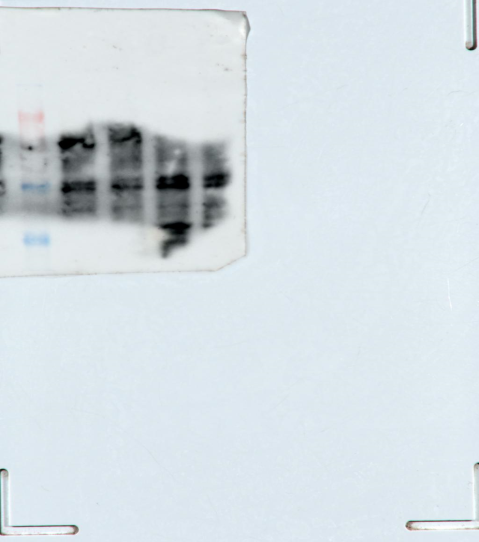


43 KDa

43 KDa

**PARP**


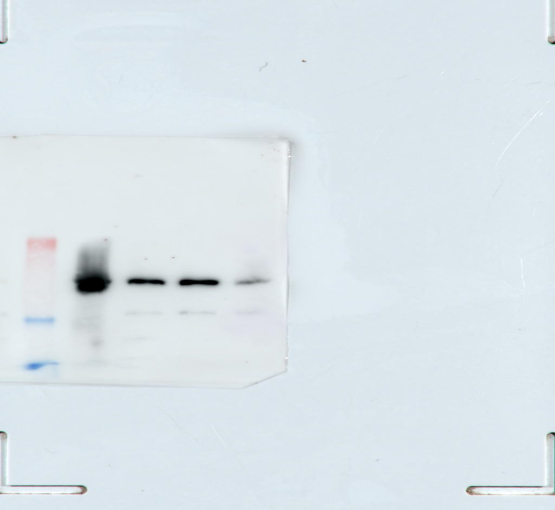

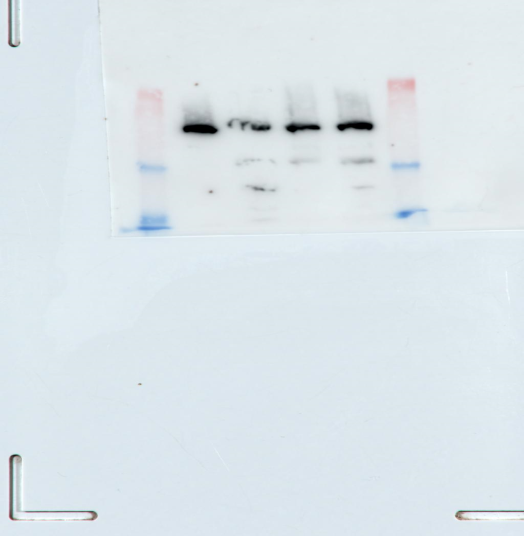

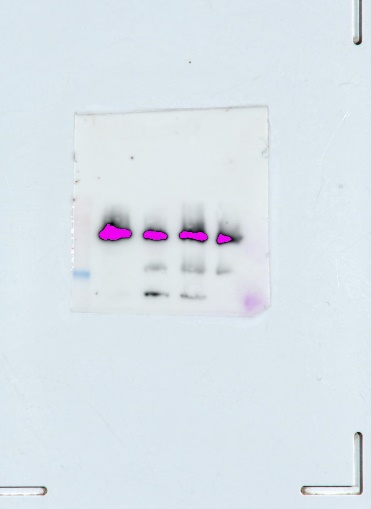


116 KDa

89 KDa

116 KDa

116 KDa

89 KDa

89 KDa

**Actin (PARP and NRF2)**


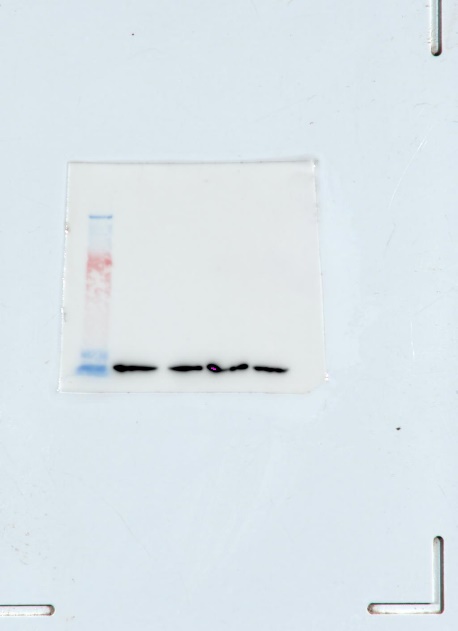

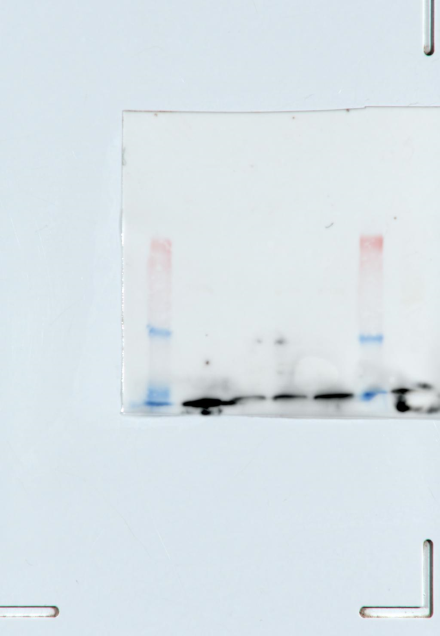

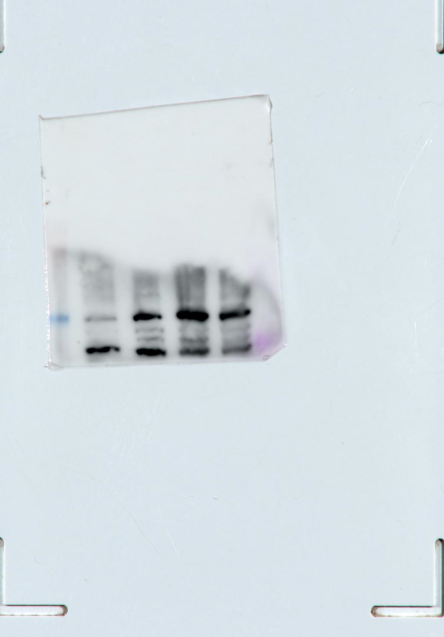


43 KDa

43 KDa

43 KDa

**NRF2**


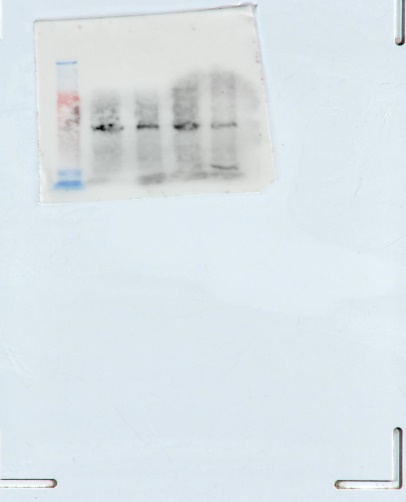

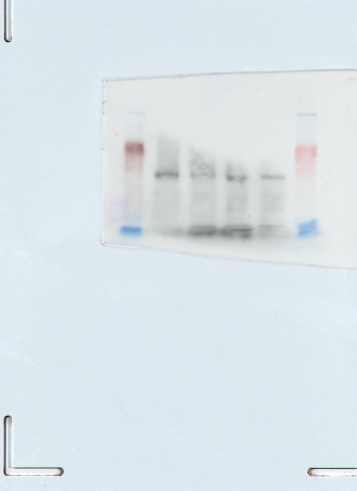

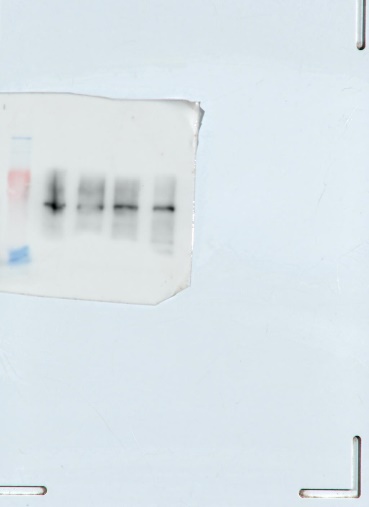


62 KDa

62 KDa
